# The spatiotemporal spread of cervical spinal cord contusion injury pathology revealed by 3D in-line phase contrast synchrotron X-ray microtomography

**DOI:** 10.1016/j.expneurol.2020.113529

**Published:** 2021-02

**Authors:** Merrick C. Strotton, Andrew J. Bodey, Kazimir Wanelik, Carl Hobbs, Christoph Rau, Elizabeth J. Bradbury

**Affiliations:** a King's College London, Wolfson Centre for Age Related Diseases, Institute of Psychiatry, Psychology & Neuroscience, Guy's Campus, London Bridge, London SE1 1UL, UK; b Diamond Light Source, Oxfordshire OX11 0DE, UK

**Keywords:** Synchrotron, Microtomography, Spinal cord injury, Neurotrauma, White matter, Gray matter, CSA, central spinal artery, DSV, dorsal spinal vein, FFPE, fixed-formalin paraffin embedded, SCI, spinal cord injury, SRμCT, synchrotron radiation microtomography.

## Abstract

Extensive structural changes occur within the spinal cord following traumatic injury. Acute tissue debris and necrotic tissue are broken down, proliferating local glia and infiltrating leukocytes remodel tissue biochemical and biophysical properties, and a chronic cavity surrounded by a scar forms at the injury epicentre. Serial-section 2D histology has traditionally assessed these features in experimental models of spinal cord injury (SCI) to measure the extent of tissue pathology and evaluate efficacy of novel therapies. However, this 2D snapshot approach overlooks slice intervening features, with accurate representation of tissue compromised by mechanical processing artefacts. 3D imaging avoids these caveats and allows full exploration of the injured tissue volume to characterise whole tissue pathology. Amongst 3D imaging modalities, Synchrotron Radiation X-ray microtomography (SRμCT) is advantageous for its speed, ability to cover large tissue volumes at high resolution, and need for minimal sample processing. Here we demonstrate how extended lengths of formalin-fixed, paraffin-embedded (FFPE) rat spinal cord can be completely imaged by SRμCT with micron resolution. Label-free contrast derived from X-ray phase interactions with low-density soft tissues, reveals spinal cord white matter, gray matter, tissue damage and vasculature, with tissue still viable for targeted 2D-histology after 3D imaging. We used SRμCT to quantify tissue pathology after a midline, cervical level (C6), 225 kDyne contusion injury over acute-to-chronic (24 h to 5 weeks) post injury time points. Quantification revealed acute tissue swelling prior to chronic atrophy across the whole imaged region (spanning 2 spinal segments above and below injury), along with rostro-caudal asymmetries in white and gray matter volume loss. 3D volumes revealed satellite damage in tissue far removed from the epicentre, and extensive rostro-caudal spread of damage through the base of the dorsal columns at 24 h post injury. This damage overlapped regions of vasogenic oedema, confirmed with subsequent histology. Tissue damage at later time points in border regions was most prominent in the dorsal columns, where it overlapped sites of damaged venous vasculature. Elaborating rostro-caudal and spatiotemporal asymmetries in reduced traumatic injury models centred on these regions may inform future treatments that seek to limit the spread of tissue pathology to these ‘at-risk’ regions.

## Highlights

• Whole rat spinal cord SRμCT tomograms (up to 20 mm length) with μm resolution
• Pathology of 3 SHAM and 24 acute-to-chronic C6 midline contusion SCIs quantified
• Rostro-caudal asymmetries in gray and white matter pathology progression
• Differences in ascending and descending dorsal column tract pathology
• Delayed rostral-caudal pathology associated with sites of venous vasculature

## Graphical abstract

**Figure:**
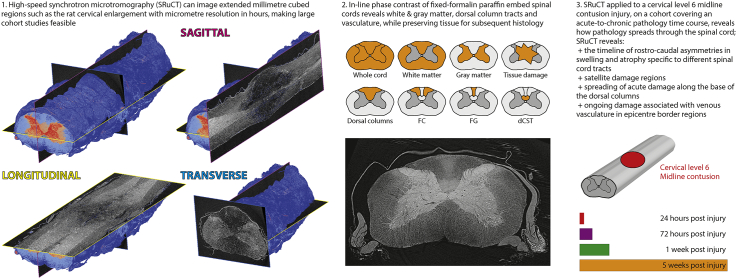

## 1 Introduction

The spinal cord undergoes extensive structural remodelling following injury (Ahuja et al. 2017; Norenberg et al. 2004). Mechanically ruptured tissue is broken down by resident and infiltrating immune cells (Donnelly and Popovich 2008; Gensel and Zhang 2015; Greenhalgh et al. 2020), extracellular matrix remodelling and scarring modifies the biochemical (Didangelos et al. 2016) and biophysical (Moeendarbary et al. 2017) properties of tissue, and a fluid filled cavity surrounded by a glial scar forms around the chronic spinal cord injury (SCI) epicentre (Ahuja et al. 2017; Bradbury and Burnside 2019). In experimental models of SCI, these features are routinely measured by 2D histology to track pathology and evaluate the efficacy of novel therapeutic agents. Such 2D tissue measurements are subject to inaccuracies that arise from mechanical processing artefacts, errors in section collection, and also misrepresentation by conscious/unconscious bias in section choice for analysis. Complete characterisation of whole tissue pathology by 3D imaging avoids these issues, improving the accuracy, reliability and ultimate utility of data.

A variety of optical and non-optical 3D imaging methodologies exist. For static, relativistic spatial imaging, *ex vivo* methods offer improved parameters over *in vivo* counterparts. For example, *ex vivo* diffusion tensor imaging (DTI) can achieve higher spatial resolution of larger tissue regions than its *in vivo* counterpart (Ellingson et al. 2008; Jirjis et al. 2013), while *ex vivo* tissue clearing overcomes the depth limitations (~1 mm *in vivo*)(Erturk et al. 2012; Helmchen and Denk 2005; Ouzounov et al. 2017) of optical imaging to reveal fluorescent markers in whole tissues, or even whole animals (Cai et al. 2019; Pan et al. 2016; Richardson and Lichtman 2015). More generally, *ex vivo* imaging avoids *in vivo* complications such as anaesthesia and sample orientation limits. Samples can also be easily stored and shared.

Synchrotron Radiation X-Ray Microtomography (SRμCT) is a quasi-non-destructive, label-free 3D imaging technique, able to derive soft tissue contrast from minimally processed formalin-fixed, paraffin-embedded (FFPE) samples (Mizutani and Suzuki 2012). It does not rely on labelling efficacy that can affect affinity fluorescent-tag imaging, nor does it require exclusive sample generation as is necessary for 3D imaging of cleared tissues. Absorption X-ray tomography has previously been used in combination with perfused radiopaque plasticising agents to improve vascular contrast for vascular segmentation in healthy central nervous system tissue and spinal thoracic level ischaemia or contusion injury models (Cao et al. 2015; Cao et al. 2017; Hu et al. 2015). However, in-line phase propagation X-ray imaging can avoid plasticising agents to instead derive FFPE soft tissue contrast through X-ray interactions with low-density tissue and edge features (*e.g.* cell-cell, axon-myelin borders) (Hu et al. 2015; Strotton et al. 2018). This unambiguously reveals macro- features including white matter and gray matter alongside vasculature, while maintaining samples for subsequent histology. A great advantage of SRμCT is that it can rapidly (~15 min/scan) image large volumes (~50 mm^3^,) at high resolution (several microns). Scan speeds make imaging multiple samples as well as concatenating multiple tomograms to cover extended regions of large tissues practical within a short time frame (*i.e.* a single synchrotron beam time). For comparison, label-free 3D imaging techniques such as *ex vivo* DTI, take ~13 h to image 2 mm thick slices through the rat spinal cord with lower resolution (Ellingson et al. 2008; Jirjis et al. 2013).

The majority of clinical SCIs impact the spinal cord cervical enlargement due to the relative exposure of the neck region (National Spinal Cord Injury Statistical Center at the University of Alabama, 2019). Rat contusion SCI models recapitulate several clinical SCI pathological hallmarks, including progressively spreading tissue damage, cavitation and formation of a fibrotic and astrocytic scar (James et al. 2011). Rat cervical level contusion injury models are therefore useful for testing injury modifying interventions in a clinically-relevant system (Burnside et al. 2018; James et al. 2015). SCI pathology progression is often sub-divided into primary (the initial mechanical trauma delivered to the spinal cord) and secondary damage (a delayed injury cascade that leads to a worsening of outcome (Ahuja et al. 2017)). Limiting the spread of secondary damage is one of the major therapeutic goals of SCI research, with an array of ‘protective’ interventions being developed (Kwon et al. 2011). The spinal segmental distribution of function means that at the cervical level, even limited reduction of damage spread could mean the difference between retaining or losing hand and arm function. A detailed 3D characterisation of how tissue damage spreads through the rat spinal cord in whole tissue samples following a cervical level contusion would improve our understanding of the spatiotemporal characteristics of spreading tissue damage after traumatic injury. This would provide important insight into understanding the basis against which to judge injury-modifying interventions and identify which aspects of injury might reasonably be targeted by treatment.

SRμCT was used to image the complete dorso-ventral and medio-lateral extent of the rat cervical enlargement along extended lengths of spinal cord (over 2 cm). 3D volumes of whole spinal cord samples spanning 2 segments above and below a midline cervical level 6 (C6) spinal contusion injury site were collected at 24 h' post injury (HPI), 72 HPI, 1 week post injury (WPI) and 5 WPI, to assess pathology over acute-to-chronic time points. Comparing tissue volume, white matter, gray matter and tissue damage between samples topologically aligned to root entry zones, confirmed hallmark pathological features, including acute cord swelling that progresses to chronic atrophy. Several rostro-caudal asymmetries in the extent and/or rate of white/gray matter volume changes above and below the injury epicentre were also revealed, which would typically be masked through normalisation approaches common to 2D histology. Panning high-resolution 3D scan volumes revealed damage spreading extensively into border tissue from the epicentre along the base of the dorsal columns by 24 HPI, and discrete satellite lesions in epicentre border tissue that appeared otherwise normal. Such features reveal the rapid spread of tissue damage through the spinal cord along gray-white matter tissue interfaces by 24 HPI. In revealing the subtleties underlying the emergence of hallmark pathological features, these datasets should guide future attempts that seek to limit damage spread through the spinal cord.

## 2 Materials & methods

### 2.1 Compliance with ethical standards

All animal study procedures were carried out in accordance with UK Home Office legislation (Scientific Procedures Act 1986) and approved by the Animal Welfare and Ethical Review Body at King's College London, where tissue was generated for this study.

### 2.2 Animal surgery and tissue collection

Cervical level 6 (C6) spinal contusion surgeries were performed on adult male Lister Hooded Rats (320 to 380 g at time of surgery; Harlan), as previously described (Burnside et al. 2018; James et al. 2015). Briefly, animals were anesthetised by intraperitoneal (i.p.) injection of a ketamine (60 mg/kg) - medetomidine (0.25 mg/kg) mix. Once no longer reflexive, an area overlying the shoulder blades was shaved and swabbed with ethanol and iodine. Animals were then transferred to a sterile field overlying a heat pad to maintain core temperature at ~36.5 °C (monitored with a rectal probe) during surgery. Muscle layers overlying spinal level C6 were then retracted and a C6 laminectomy performed to expose the underlying spinal cord. A 225 kDyne midline contusion was delivered to the exposed cord using a 2.5 mm diameter probe (Precision Systems Instrumentation, USA). Animal muscle and skin layers were then re-sutured, and animals transferred to a holding incubator until anaesthesia was reversed 1.5 h after induction. All animals received 3 days of post-operative saline (3–5 mL) and analgesia (carprofen; 5 mg/kg) and a one-week course (or until end-point) of antibiotics (baytril; 0.05 mg/kg) during recovery. In total, 27 animals were used to generate the pathology time course data (5 WPI, *n* = 6; 1 WPI, n = 6; 72 HPI, n = 6; 24 HPI, n = 6; SHAM, *n* = 3). SHAMS received only a laminectomy.

At the appropriate time after injury, animals were sacrificed with an overdose of sodium pentobarbital (Euthatal, 80 mg/kg, i.p.). They were then transcardially perfused with PBS heparin (0.01%), followed by 400 mL of 4% PFA (0.1 M PB, pH 7.4). Tissues were then post-fixed overnight in the same fixative solution before being rinsed in 2 × 24-h PBS (pH 7.4) washes.

### 2.3 *Ex vivo* Lugol's iodine staining and paraffin wax embedding

Fixed whole spinal cords were stained by incubation in an excess of 25% Lugol's Iodine (LI) solution for 48 h (Metscher 2009; Strotton et al. 2018). Tissues were then agitated in 2 × 24-h PBS washes to remove excess iodine. For paraffin embedding, tissues were dehydrated through 8-h incubations in 30, 50 and 70% ethanol with agitation before being transferred to a paraffin wax processing station (Leica TP1020). Here, tissues were entirely dehydrated through 2-h washes in 90% ethanol and 3 × 100% ethanol. Tissues were then cleared through 2-h washes in Xylene-ethanol 1:1 and 3 x Xylene, before being infiltrated by wax with 2 × 2-h paraffin wax incubations at 60 °C. Tissues were then transferred to a paraffin wax embedding station and cast in custom silicon moulds, ensuring the spinal cord was centrally embedded and surrounded by ~3 mm of wax on all sides. Once embedded, tissues were stored at room temperature until tomographic imaging.

### 2.4 Synchrotron microtomography imaging

Microtomography was performed at the Diamond-Manchester Imaging Branchline I13-2 of the Diamond Light Source Synchrotron (Oxfordshire, United Kingdom)(Pešić et al. 2013; Rau et al. 2011) using previously optimised imaging conditions (Strotton et al. 2018). Briefly, in-line phase contrast imaging was performed with a sample-to-detector propagation distance of 160 mm and a filtered pink X-ray beam with weighted mean energy of 27 keV.

Samples were mounted and vertically aligned to the tomography rotational axis on a HUBER 1002 manual goniometer (HUBER Diffraktionstechnik GmbH & Co. KG, Germany). This was mounted on an Aerotech ABRT-260 (Aerotech Inc., USA) rotation stage. 4× compound magnification imaging was conducted with a pco.edge 5.5 (PCO AG, Germany) detector (sCMOS sensor of 2560 × 2160 pixels), by using a 2× objective coupled to a 500 μm CdWO_4_ scintillator, mounted ahead of a second 2× lens. This gave a field of view (FoV) of 4.2 × 3.5 mm (effective pixel size 1.625 μm) that could encompass the entire lateral and dorso-ventral width and depth of the rat spinal cord cervical enlargement. 40 flat- and 40 dark-field images were collected before 6001 equally-spaced sample projections (step-size 0.03°) across 180° of continuous constant-speed rotation. Exposure times of 0.12 s (~15 min/scanset) maximised use of the detector dynamic range without saturation.

After sample alignment, a custom script for automatic collection of concatenated datasets performed automated stage raising of 3.43 mm. A second scanset with the same imaging conditions was then collected, overlapping the former by 2%. This overlap aided later alignment and tomogram concatenation. For each spinal cord sample, at least 3 sequential datasets spanning 10.29 mm of spinal cord (~50 min total scan time) were collected. For SHAM spinal cord samples, between 5 and 9 concatenated datasets were collected.

### 2.5 Tomographic reconstruction of spinal cords

To derive high quality tomographic reconstructions, data were filtered to remove artefacts and correct distortions introduced by the imaging system (Strotton et al. 2018). Briefly, sample projections were normalised (flat- and dark-field corrected) and corrected for radial lens distortion (Titarenko et al. 2010). Sinograms for reconstruction were ring artefact corrected (Titarenko 2016). Phase retrieval was then conducted on projections using a Paganin filter (Paganin et al. 2002) set to the peak X-ray energy of 27 keV, effective pixel size 1.6125 μm, propagation distance 160 mm and a *δ*/β ratio of 3 (optimised in (Strotton et al. 2018)). Tomographic slices were then generated *via* the filtered back projection algorithm (FBP)(Atwood et al. 2015; Kak and Slaney 2001; Wadeson and Basham 2016). Tomograms were concatenated by manually removing overlapping tomographic slices in ImageJ (NIH, USA)(Schneider et al. 2012).

### 2.6 Histology and immunolabelling

For histology, removing iodine from tissue helped sections adhere to glass slides. To remove iodine, tissues were dewaxed by heating to 60 °C and then passed through 2 × 8-h xylene washes with agitation at room temperature. Tissues were then rehydrated through 8-h incubations of ethanol:ddH_2_O at 100, 70, 50, 30 and 0%. Next, iodine was then removed from tissue by incubating in a 5% (*w*/*v*) solution of sodium thiosulphate overnight (Strotton et al. 2018). Tissues were then dehydrated and re-embedded in paraffin wax as above. 7 μm transverse sections cut with a microtome were collected onto positively charged glass slides for staining.

For haematoxylin and eosin staining, sections were first dewaxed in an oven for 60 min at 60 °C, then washed in 2 × 10-min xylene incubations and rinsed 4 × 30-s in 100% ethanol. Slides were then rinsed in ddH_2_O and placed in haemalum for 5 min. Slides were rinsed in running cold water to remove excess haemalum, before dipping 5 times in 0.5% HCl in 70% ethanol to differentiate. Sections were then rinsed in water and counterstained with 0.5% eosin for 10 min. Excess stain was removed in water, then stained sections were dried onto slides by placing in an oven for 60 min at 60 °C. Sections were then cleared in xylene for 5 min before cover-slipping in DPX mounting media overnight to dry.

For Nissl staining, sections were dewaxed and xylene cleared as for H&E. Sections were rehydrated, placed in cresyl violet (5% w/v in ddH2O) for 10 min, rinsed in ddH2O to remove excess cresyl violet, then dried onto slides in an oven for 60 min at 60 °C. Sections were then cleared in xylene for 5 min before cover-slipping in DPX mounting media overnight to dry.

For immunolabelling of CD68 positive cells and IgG, sections were dried onto slides overnight at room temperature, then heated to 60 °C for 60 min to dewax. Slides were placed in 2 × 10-min xylene incubations, then rinsed 4 × 30-s in 100% ethanol. For antigen retrieval, tissues were then incubated in 3% hydrogen peroxide for 10 min, before placing slides in a pressure cooker at 90 °C filled with 1% citric acid (pH 6.04) for 5 min. Slides were then rinsed in water and blocked in 1% goat serum (in TBS pH 7.6) for 1-h, followed by biotinylated goat anti-rat IgG (Vectorlabs, #BA-9401) at 1:1000 in block solution overnight. For CD68 staining, slides were blocked in 1% bovine serum (in TBS pH 7.6) for 1 h, followed by mouse anti-rat CD68 (Abcam, #ab31630) at 1:500 in block solution overnight. Slides were then washed in 2 × 10-min TBS washes, before applying StreptABC-HRP (Vectorlabs, #PK-6100) to slides for 30 min. Slides were rinsed with 2 × 2 min TBS washes, then placed in 10% ImmPact DAB solution (3,3- diaminobenzidine tetrahydrochloride; Vectorlabs, #SK-4105) for ~1-min. Slides were then rinsed in water before counterstaining in haemalum as detailed above. Slides were dehydrated for 60 min at 60 °C, placed in xylene to clear for 5 min and cover-slipped in DPX mounting media at room temperature overnight to dry. All slides were imaged under brightfield illumination using a Zeiss AxioCam.

### 2.7 Morphological assessments and statistics

All tomogram image processing, segmentation and analyses were performed using ImageJ (NIH, USA)(Schneider et al. 2012). For measuring macro features, the complete scanset for each sample was compressed to 16-bit and median binned 2 × 2 × 2 (to an effective voxel size of 3.2 μm). This reduced salt-and-pepper noise and – together with the bit-depth reduction – reduced file size for faster image processing (2^4^ times smaller files) during analysis. Spinal cord area, tissue damage (defined as tissue debris and cavity), gray matter, white matter and dorsal columns were then manually annotated by a blinded investigator in 3.2 μm thick serial sections extracted *in silico* at regular 403.2 μm (every 126th slice) intervals through the entire imaged volume. Data, statistical comparisons and graphs were generated in GraphPad Prism (v7.0a). All statistical outputs for 2-way ANOVA (Fig. 4.A, E, I, M, Q) and 1-way ANOVA (Fig. 4.B-D, F—H, J-L, N—P, R-T) are available in index matched Supplementary Tables (Table.S1.A-T) available in Supplementary file 7. Summary graphs were generated in orthogonal planes by re-slicing data within ImageJ, with average (mean), maximum and minimum intensity projections of multiple 1.6 μm thick *in silico* slices, as indicated in figures. In Fig. 6, tomograms were matched to histological images by re-slicing and thickness adjusting in IMOD (Kremer et al. 1996).

Cell size-frequency distribution was performed on Nissl stained spinal cord sections from rostral and caudal border regions of SHAM (*n* = 3), 24 HPI (*n* = 5), 72 HPI (n = 5) and 1 WPI (n = 5) animals in a blinded manner. For each animal, 1 rostral and 1 caudal section corresponding to the shaded rostral and caudal border regions in Fig. 4.A were selected for analysis (36 sections total; 1 rostral and 1 caudal section from 18 animals). On these 36 sections, an Ilastik (v1.1.3) (Berg et al. 2019) pixel classifier was trained to recognise Nissl stained features within a manually traced gray matter mask (Sup.Fig. 2.A,B). Using CellProfiler (v4.0) (McQuin et al. 2018), Nissl features were segmented to individual objects (Sup.Fig. 2.C) that were then filtered to Nissl positive cells containing a manually identified nucleus (Sup.Fig. 2.D; 6871 neurons across all sections). Graphs and statistical comparisons of the Nissl positive cell size-frequency distributions were generated in Python using the Seaborn and SciPy packages, respectively.

### 2.8 Behavioural assessments

Motor and sensory forelimb behaviours were assessed in a separate cohort of age and weight matched Lister hooded rats that received the same contusion injury as the synchrotron pathology group (*n* = 8). These animals were assessed in multiple behavioural tasks, including the forelimb locomotor scale (FLS) (Singh et al. 2014), grip strength (Meyer et al. 1979), horizontal ladder (Metz and Whishaw 2009) and sticker sensation latency task (Komotar et al. 2007). All behaviours were scored at 5, 7, 10, 14, 21, 28 and 35 DPI. FLS, grip strength and horizontal ladder were also scored at 3 DPI. Three horizontal ladder and grip strength baseline scores were collected after 2 weeks of habituation training at −1, −3 and − 5 DPI with the average of these taken as baseline. FLS and sticker sensation baseline scores were collected once (−1 DPI), to maintain task novelty.

For grip strength, rats were supported by their hindlimbs and ventral side, then withdrawn from a grip strength bar (Linton instruments, U.K.) to which they reflexively grip before relinquishing the bar to give a force readout. On each test day, the maximum reading from 10 passes over the bar was recorded at 3 intervals separated by at least 1 h. The mean of these 3 recordings was the grip strength score for that day.

For horizontal ladder, rats were trained to run across a 1 m ladder with unevenly spaced rungs for 10 min, 5 days/week, 2 weeks prior to baselining. Recordings were made (Sony DCR-SX30E Handycam) and later forelimb slips scored in videos of 3 ladder crossings. The number of forelimb slips were scored as a percentage of total forelimb steps.

For sticker sensation, a circular sticker (ø = 8 mm, Rymans, U.K.) was applied to the plantar surface of both forepaws and the rat placed in a clear Perspex testing chamber (ø = 45 cm). The time to acknowledge/sense the sticker (apparent as a hand to mouth movement or wrist flick) on either paw (whichever first) was recorded as sticker sensation latency. If this exceeded 60 s, a score of 60 s was assigned. Animals were kept in the chamber for at least 120 s regardless of how early sticker sensation was detected.

## 3 Results

### 3.1 Concatenated tomograms and volume quantification of the entire intact rat cervical spinal cord

Using previously optimised conditions to balance data quality against acceptable time penalties (Strotton et al. 2018), 6001 projection SRμCT scans were collected with the full array of a pco.edge 5.5 detector at 4× magnification (4.2 × 3.5 mm, pixel size 1.6 μm) in ~12 min. This FoV encompassed the widest part of the rat spinal cord cervical enlargement. By raising spinal cords along their longitudinal axis through this FoV, extended lengths of spinal cord could be imaged in sequential, concatenated scans.

**Fig. 1:**
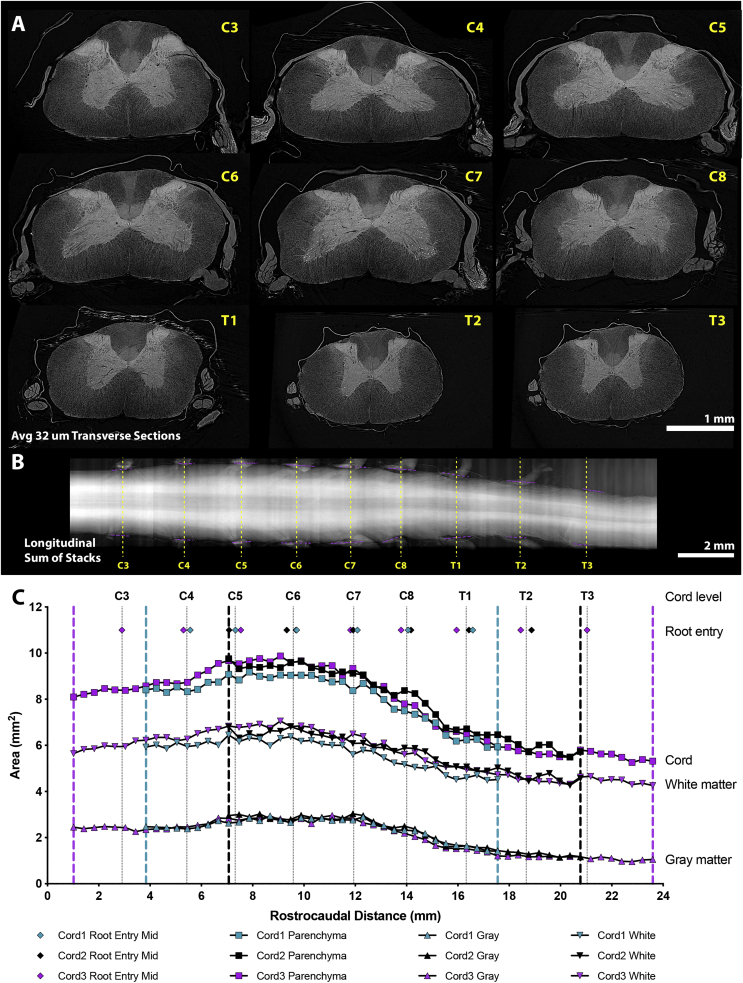
Spinal cord, white and gray matter changes through the SHAM spinal cord cervical enlargement. (A) 32 μm thick average transverse slices of Cord3 from each spinal cord segmental level within a scan series spanning the cervical spinal cord (from C3-C8) and 3 thoracic segments (T1-T3). (B) A longitudinal summed projection through the entire dorso-ventral axis of the spinal cord, where the root entry level on each side of the cord is marked (purple dashed plot). The midpoint of these is taken as the root entry point (yellow dashed line) at each cord level (from which the transverse slices in (A) are taken). This image is aligned to plot (C) which shows all three spinal cords (blue Cord1, black Cord2, purple Cord3) aligned to their root entry points (colour matched diamond symbols). The raw area (mm^2^) of total spinal cord, white matter, and gray matter were measured in transverse sections at 403.2 μm intervals. The rostro-caudal extent of each of the 3 SHAM spinal cord scans are demarcated by vertical dashed lines (blue Cord1, black Cord2, purple Cord3). Cords were aligned at their root entry point, and total area, white matter and gray matter area can be seen to align consequently.

Five concatenated scans covering the cervical enlargement of 2 SHAM rat spinal cords (Cord1 and Cord2, scans spanning 13.71 mm of spinal cord) were performed in ~80 min, while 9 concatenated scans (Cord3, spanning 22.58 mm of spinal cord, Supplementary Video 1) of a third SHAM spinal cord were performed in ~150 min (Fig. 1). Transverse slices generated *in silico* at the midpoint of the dorsal root entry zone (identified in the longitudinal plane, Fig. 1.B), demonstrate the characteristic segmental anatomy along the rostro-caudal axis from cervical level 3 (C3), to thoracic level 2 (T2) (Fig. 1.A). Measuring white and gray matter area in transverse slices every 403.2 μm (every 126th slice) within the 3 SHAM spinal cords and then aligning these to root entry zones, aligns spinal cord, white and gray matter area measurements. The narrow range of these area measurements highlights the accuracy of measuring gross cord features by SRμCT (Fig. 1.C). Thus, SRμCT enables accurate and reproducible measurements of white and gray matter volume at defined levels of the spinal cord. We next applied SRμCT to study the characteristics of progressive tissue pathology after traumatic SCI.

### 3.2 Multi-plane 3D exploration of spinal cord injury pathology

**Fig. 2:**
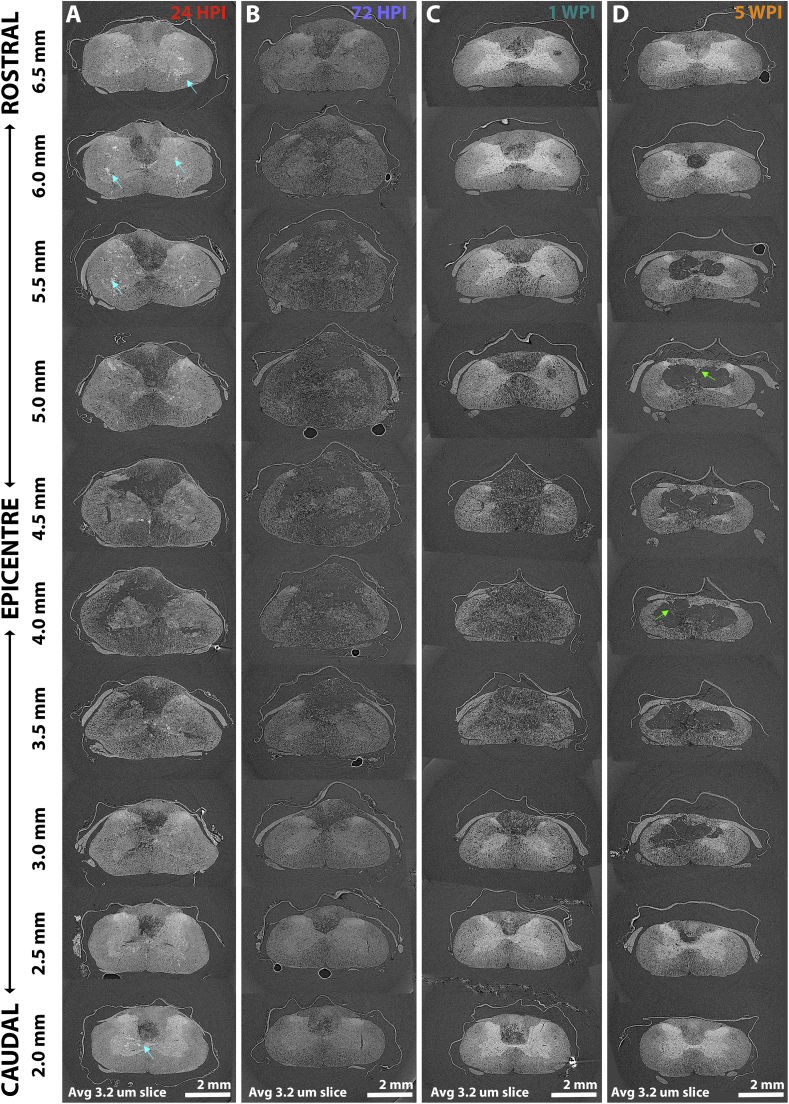
Serial transverse projections through a C6 midline contusion epicentre at multiple time points after injury. (A) 24-h after a dorsally delivered contusion injury, immediate damage is noted within the injury epicentre. The dorsal and ventral columns are particularly damaged, but fragmented ‘gray matter’ can still be recognised. Damage extending rostral and caudal to the injury is mainly within the dorsal column. Satellite regions of damage (high contrast regions indicated by cyan arrows) appear in tissue surrounding the injury epicentre, though are not apparent at later time points. (B) At 72 HPI, gross damage predominates. ‘Gray matter’ regions can no longer be discerned and the extent of damaged tissue has spread relative to 24 HPI. Swelling of the cord following the initial contusion is apparent at 24 and 72 HPI at the epicentre (~4.5 mm). (C) Swelling has subsided by 1 WPI. Central damage leaves only a thin rim of tissue intact at the epicentre, with most of the central region being filled with debris. Moving rostro-caudal, damage is predominantly in the dorsal columns. (D) At 5 WPI, a cavity has formed in the epicentre spanned by tissue bridges (green arrows, also seen in the longitudinal plane in Fig. 3). These tomograms are quantified in Fig. 4.

**Fig. 3:**
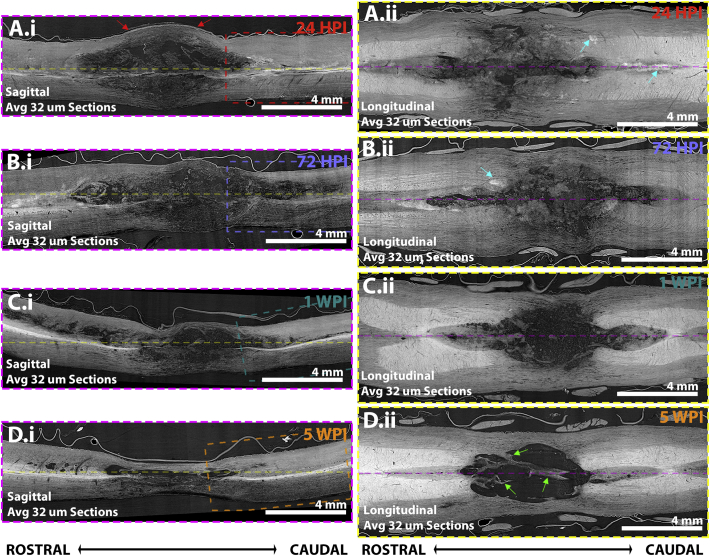
Matched mid-sagittal and mid-longitudinal planes showing the emergence of acute-to-chronic tissue damage. The same spinal cords presented as transverse sections in Fig. 2 are here projected along sagittal (i) and longitudinal (ii) planes. (A) At 24 HPI, damage at the injury site spans the width of the spinal cord, spreading centrally in rostro-caudal directions. The sagittal plane demonstrates local swelling of tissue in contact with the overlying dura matter (red arrows). The longitudinal plane also highlights satellite tissue damage regions (cyan arrows) separate from the epicentre. (B) At 72 HPI, swelling has subsided, but the injury extent has spread. (C) At 1 WPI the injury does not appear to have spread further, but debris appears more fragmented than at earlier time points. (D) By 5 WPI, tissue atrophy extending rostro-caudally to the injury epicentre is apparent, with obvious contraction of the injury epicentre. Yellow dashed lines in sagittal sections and pink dash lines in longitudinal sections indicate the relationship between sagittal and longitudinal planes.

**Fig. 4:**
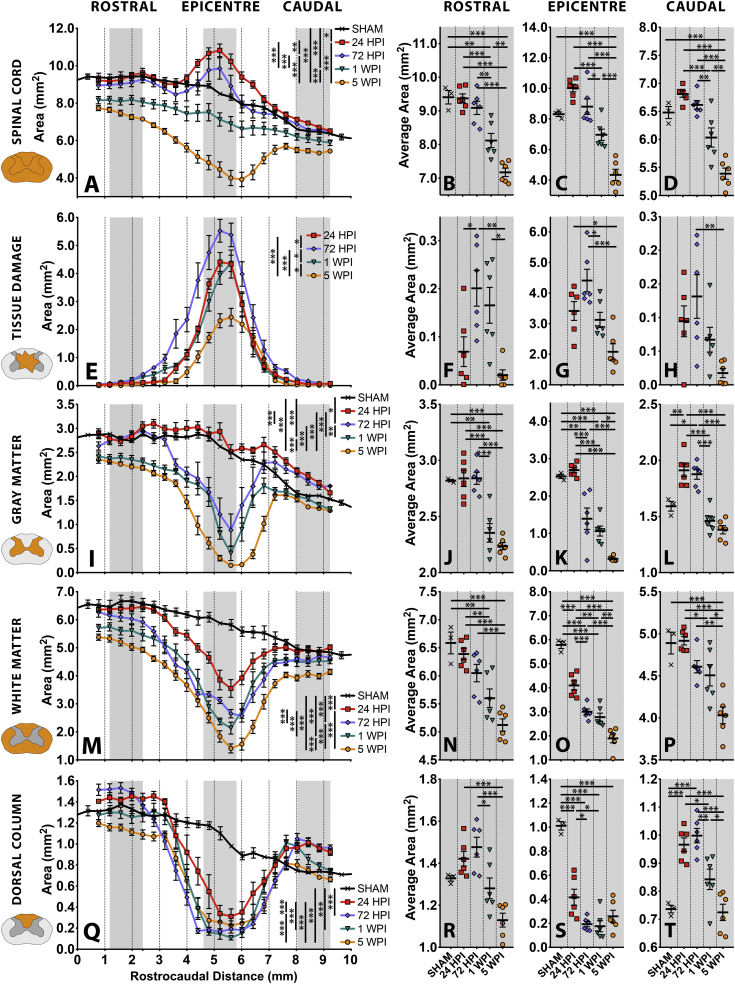
Quantifying acute-to-chronic cord area, tissue damage, gray matter, white matter and dorsal column area changes following a C6 midline contusion. (A-D) Spinal cord area (*E*-H) Tissue damage (I-L) Gray matter (M-P) White matter and (Q-T) Dorsal column area changes after injury. (A, E, I, M, Q) Area measured within transverse sections measured at 403.2 μm intervals spanning an 8.47 mm length of rat spinal cord for naïve (*n* = 3) and acute to chronic post-injury time points (*n* = 6/group). Comparisons made with two-way ANOVA with Tukey's post-hoc multiple comparisons. (B, F, J, N, R) Rostral, (C, G, K, O, S) epicentre and (D, H, L, P, T) caudal changes occurring in shade-matched regions of (A, E, I, M, Q). Each point represents an individual animal. Comparison made with one-way ANOVA with Tukey's post-hoc multiple comparisons. All graphs mean ± SEM. Significance * < 0.05, ** < 0.01, *** < 0.001. All statistical comparisons available in index matched supplementary tables (Supplementary table 1).

To measure acute-to-chronic SCI pathology progression, SRμCT scans of rat C6, 225 kDyne midline contusion SCI epicentres were collected at 24 HPI, 72 HPI, 1 WPI and 5 WPI. Three concatenated scans (~45 min acquisition) were performed per sample, within which 8.43 mm of spinal cord overlapped about the epicentre of all scans. Spinal cord volumes could be re-sliced in any plane, so transverse (Fig. 2), sagittal (Fig. 3, left panels) and longitudinal (Fig. 3, right panels) planes could be extracted from the same spinal cord volumes to emphasise and reveal different aspects of tissue pathology. Panning the complete 3D volume then helped verify anatomical features and explore how contusion pathology spreads through the spinal cord (quantified in Fig. 4).

Serial transverse image slices at 0.5 mm intervals (Fig. 2) show mass tissue necrosis at early time points, particularly around the injury epicentre and surrounding penumbra. This was evident at 24 HPI (Fig. 2.A, Supplementary Video 2) and most prominent at 72 HPI (Fig. 2.B, Supplementary Video 3). Areas of ongoing necrosis were still apparent at 1 WPI but tissue pathology was progressing into cavity formation, with areas of cavitation beginning to emerge together with signs of overall cord shrinkage (Fig. 2.C, Supplementary Video 4). By 5 WPI, large areas of central cavitation and significant cord shrinkage were apparent (Fig. 2.D, Supplementary Video 5). Longitudinal (Fig. 3, left panels) and sagittal (Fig. 3, right panels) plane projections of those SCI volumes shown in the transverse plane support these observations and help to discern additional features of pathology progression. Acute cord swelling for instance was particularly evident at 24 HPI in the sagittal view, where the cord parenchyma was in close contact with surrounding dura matter (red arrows, Fig. 3.A.i) that was displaced from the spinal cord at later time points. Steady breakdown of necrotic tissue at 72 HPI (Fig. 3.B.i) that proceeds to cavitation at 1 WPI (Fig. 3.C.i) and cavitation alongside tissue shrinkage was also apparent in the sagittal plane by 5 WPI (Fig. 3.D.i). This pattern could also be seen in longitudinal projections (Fig. 3, right panels). Longitudinal projections also helped to highlight multiple regions of satellite damage ~10 μm in diameter scattered across the rostro-caudal parenchyma relative to the epicentre (cyan arrows in Fig. 2.A, Fig. 3.A.ii). These are reduced in number, but still present by 72 HPI (Fig. 3.B.ii). Satellite damage foci may represent the origin of discrete cysts seen clinically at chronic time points in regions far removed from the SCI epicentre (Bunge et al. 1993; Bunge et al. 1997). Necrotic tissue and debris gives way to a single central cavity that begins to emerge by 1 WPI (Fig. 2.C.ii). Panning 3D tissue volumes confirms that the large epicentre cavity formed by 5 WPI is consistently spanned by tissue bridges that enter/exit the surrounding tissue (green arrows, Fig. 2.D, Fig. 3.D.ii). Differences can be compared between time points in aligned transverse fly throughs of those samples presented in Fig. 2 (Supplementary Video 6).

### 3.3 Quantifying acute to chronic spinal cord injury pathology in the rat cervical level contusion injury model

Most studies assessing SCI tissue pathology approximate a representation of the overall spinal volume by assessing serial transverse sections that span the injury. Measurements of features within these sections are often then normalised to total cord cross sectional area (compensating for distortion acquired during mechanical processing) and compared after alignment to the injury epicentre (often defined as that section with the greatest degree of tissue damage following injury) (Chen et al. 2016; James et al. 2011; Noble and Wrathall 1985). With full 3D scans, it was instead possible to align cords to root entry zones and compare *raw* feature measurements. Unambiguous macro spinal cord features including total spinal cord area (Fig. 4.A), tissue damage (debris + necrosis, Fig. 4.E), gray matter (Fig. 4.I), white matter (Fig. 4.M) and dorsal column (Fig. 4.Q) area, were quantified within transverse sections extracted *in silico* from 3D scans at regular 403.2 μm intervals. The mean area of 4 transverse slices (spanning 1612.8 μm length) at the epicentre (Fig. 4.C, G, K, O, S) and at an equal distance rostral (Fig. 4.B, F, J, N, R) and caudal (Fig. 4.D, H, L, P, T) to the epicentre were also calculated to highlight pathology development in regions bordering the injury.

Spinal cord area significantly changed over the acute to chronic time course (F (4, 22) = 39.64, *p* < 0.0001; Two Way RM-ANOVA with Tukey's post-hoc; Fig. 4.A, Sup1.A), with acute tissue swelling at 24 HPI, subsiding towards a SHAM ‘normal’ volume by 72 HPI. This then progressed to significant atrophy at 1 & 5 WPI. At 24 HPI, acute swelling mainly occurred within the injury epicentre (Fig. 4.C) and caudal (Fig. 4.D) region. Significant atrophy first appeared rostral to injury at 1 WPI (Fig. 4.B, Table.S.1.B), but by 5 WPI, atrophy reached significant levels across rostral, epicentre (Fig. 4.C, Table.S.1.C) and caudal (Fig. 4.D, Table.S.1.D) regions. By 5 WPI, the most extensive volume loss occurred at the epicentre (47.1% volume reduction relative to SHAMs), with a more moderate volume loss rostral (16.8% relative to SHAM) and caudal (23.8% relative to SHAM) to injury. To characterise these tissue volume changes in more detail, tissue damage, gray matter, white matter and dorsal column area were quantified in the same transverse sections.

Damage within the spinal cord proceeds from acute tissue necrosis and debris formation to extensive cavitation over the acute-to-chronic period. However, these features are difficult to differentiate until a defined cavity forms at 5 WPI, so they were combined and termed tissue damage for quantification. Tissue damage changes significantly across injury time points (F (3,20) = 14.72, *p* < 0.0001; Two Way RM-ANOVA with Tukey's post-hoc; Fig. 4.E, Table.S.1.E). The increase in damage from 24 to 72 HPI is largely due to the ongoing necrosis and rapid breakdown of gray matter (which was apparent at 24 HPI and severe at 72 HPI; Fig. 2A,B), with a slight reduction in tissue damage from 72 HPI to 1 WPI. Further reduction in tissue damage area from 1 to 5 WPI, likely reflects the change from regions of central necrotic tissue to areas of cavitation, alongside significant cord atrophy (cord atrophy is first detected at 1 WPI, progressing further by 5 WPI). This pattern is similar across rostral (Fig. 4.F, Table.S.1.F), epicentre (Fig. 4.G, Table.S.1.G) and caudal (Fig. 4.H, Table.S.1.H) regions. Tissue damage in rostro-caudal regions was mainly observed around the base of the dorsal columns (Fig. 2, Fig. 3).

There were significant changes in gray matter volume over the 5-week time course (F (4, 22) = 49.44, *p* < 0.0001; Two Way RM-ANOVA with Tukey's post-hoc; Fig. 4.I, Table.S.1.I). The most pronounced shift was a 44.9% loss of epicentre gray matter at 72 HPI relative to SHAM, which proceeded to an 87.3% loss by 5 WPI (Fig. 4.K, Table.S.1.K). Gray matter loss was relatively delayed rostral to injury, not reaching significant loss until 1 WPI, before proceeding to 20.7% loss by 5 WPI (Fig. 4.J, Table.S.1.J). Caudally, there was a significant *increase* in gray matter volume of 20.1% and 18.0% at 24 and 72 HPI, respectively (Fig. 4.L, Table.S.1. L).

White matter volume also significantly varies over the time course (F (4, 22) = 35.96, p < 0.0001; Two Way RM-ANOVA with Tukey's post-hoc; Fig. 4.M, Table.S.1.M). Unlike the precipitous loss of gray matter at acute time points, there is a continued loss of white matter across the studied time course. Significant epicentre white matter loss of 29.1% has occurred by 24 HPI, proceeding to a 67.2% loss by 5 WPI (Fig. 4.O, Table.S.1.O). This is less than epicentre gray matter loss at 5 WPI, indicating greater sparing of white matter tissue at the epicentre than gray matter. Rostral (Fig. 4.N, Table.S.1.N) and caudal (Fig. 4.P, Table.S.1.P) white matter show a similar pattern of gradual volume loss, reaching a 13.1% and 22.7% reduction by 5 WPI, respectively. Loss of a similar white matter volume between 72 HPI to 1 WPI, and from 1 WPI to 5 WPI, indicates white matter loss has not slowed by 5 WPI.

To better define white matter changes after injury, dorsal column white matter was quantified separately. Significant changes in dorsal column volume occurred across the time course (F (4, 22) = 14.13, p < 0.0001; Two Way RM-ANOVA with Tukey's post-hoc; Fig. 4.Q, Table.S.1.Q), with extensive loss of dorsal column tissue at the epicentre as soon as 24 HPI, plateauing to a loss of ~80% by 1 WPI (Fig. 4.S, Table.S.1.S). Rostral and caudal dorsal columns demonstrated distinct patterns of volume change. No significant change occurred in the rostral dorsal column volume relative to SHAM (Fig. 4.R, Table.S.1.R), though caudal to injury there was a significant dorsal column swelling of 31.1% and 35.1% at 24 and 72 HPI, respectively (Fig. 4.T, Table.S.1.T).

**Fig. 5:**
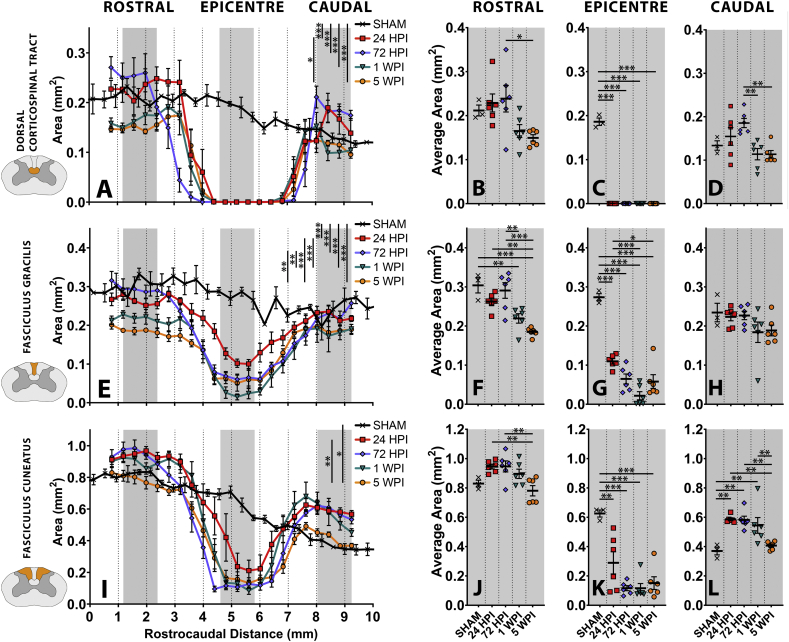
Quantifying dorsal corticospinal tract, fasciculus gracilis and fasciculus cuneatus area changes following a C6 midline contusion. The dorsal column from Fig. 4.Q was divided into (A-D) Dorsal corticospinal tract (E-H) Fasciculus gracilis and (I-L) Fasciculus cuneatus. (A, E, I) Area measured within transverse sections measured at 403.2 μm intervals spanning an 8.47 mm length of rat spinal cord for naïve (n = 3) and acute to chronic post-injury time points (n = 6/group). Comparisons made with two-way ANOVA with Tukey's post-hoc multiple comparisons. (B, F, J) Rostral, (C, G, K) epicentre and (D, H, L) caudal changes occurring in shade-matched regions of (A, E, I). Each point represents an individual animal. Comparison made with one-way ANOVA with Tukey's post-hoc multiple comparisons. All graphs mean ± SEM. Significance * < 0.05, ** < 0.01, *** < 0.001. All statistical comparisons available in index matched supplementary tables (Supplementary table 2).

The rat dorsal column is comprised of the descending dorsal corticospinal tract (dCST), ascending fasciculus gracilis (FG) and (lateral to the FG) the ascending fasciculus cuneatus (FC). The different axon types, axon density, and degree of axon myelination in these tracts, give rise to density differences that produce CT contrast suitable to quantify these tracts separately and assess their contribution to rostro-caudal volume changes within the dorsal column (Fig. 5).

Significant changes are seen in dCST volumes across the time course (F (4, 22) = 20.62, p < 0.0001; Two Way RM-ANOVA with Tukey's post-hoc; Fig. 5.A, Table.S.2.A), with complete loss of dCST at the injury epicentre at 24 HPI and all subsequent time points (Fig. 5.C, Table.S.2.C). In border regions, the only significant changes across the pathology time course were a volume reduction in the rostral dCST from 72 HPI to 1 WPI (Fig. 5.B, Table.S.2.B), and a similar reduction in caudal dCST volume from 72 HPI to 1 WPI (Fig. 5.D, Table.S.2.D).

A significant volume change in both the FG (F (4, 22) = 34.11, p < 0.0001; Two Way RM-ANOVA with Tukey's post-hoc; Fig. 5.E, Table.S.2.E) and FC (F (4, 22) = 6.52, *p* = 0.0013; Two Way RM-ANOVA with Tukey's post-hoc; Fig. 5.I, Table.S.2.I) was observed over the pathology time course. A similar pattern of FG (Fig. 5.G) and FC (Fig. 5.K) volume loss occurs at the epicentre, with 59.9% and 53.7% lost relative to SHAM respectively by 24 HPI. This increased to a 78.8% and 75.8% FG and FC loss respectively (relative to SHAM) by 5 WPI.

FG and FC volume changes differ rostral and caudal to injury over the pathology time course. Rostral to injury, a steady and significant loss of FG volume, amounted to a 39.2% loss relative to SHAM by 5 WPI (Fig. 5.F). However, rostral FC levels were relatively consistent, with only a 5.9% loss relative to SHAM by 5 WPI (Fig. 5.J). Caudal to injury, there was no significant change in FG volume across the pathology time course (F (4,22) = 1.733, *p* = 0.1787; Fig. 5.H, Table S2·H). However, there was a significant change in caudal FC volume (F (4,22) = 9.218, *p* = 0.0002; Fig. 5.L, Table.S2.L), including 58.5% volume gain relative to SHAM at 24 HPI, maintained to 72 HPI, before subsiding to ~10% swelling relative to SHAM by 5 WPI (Fig. 5.L).

### 3.4 Acute micro-fractures spread rostro-caudally along the base of the dorsal columns and align with the border of later chronic pathology

**Fig. 6:**
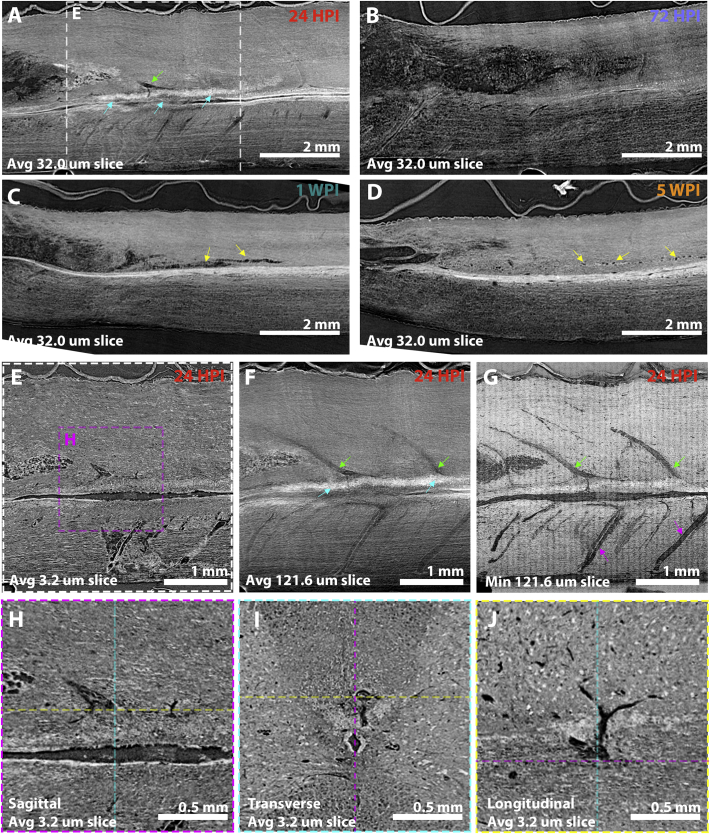
Acute spread of injury along the base of the dorsal columns and associated vasculature. (A-D) Sagittal projections of insets from Fig. 3. (A) At 24 HPI, focal damage (white high contrast signal areas, cyan arrows) is located just dorsal to the central canal. This region is local to an area at the base of a dorsal spinal vein (green arrow) as shown in E-J. (B) By 72 HPI, acute damage has been replaced by gross tissue damage that begins to collapse by (C) 1 WPI, contributing to overall tissue atrophy. (D) By 5 WPI, caudal damage appears relatively limited to focal dorsal column lesions (yellow arrows), with gross damage replaced by an overall tissue atrophy (see quantification in Fig. 4). (E-G) Different tissue thickness and intensity projections at 24 HPI from the inset in A) highlight caudal tissue damage and local vasculature. (E) A single 3.2 μm thick slice, (F) an average intensity projection of a 121.6 μm thick mid-sagittal section highlights a white band corresponding to a focal area of damage (cyan arrows) just dorsal to the central cord. This appears at the base of the large diameter dorsal spinal veins (green arrows) better highlighted in (F), a minimum intensity projection of the same 121.6 μm thick section. Fine vasculature associated to dorsal spinal veins (DSVs, green arrows) branches into the high intensity signal (cyan arrows) area of tissue damage. Central spinal arteries (CSAs, pink arrows) projecting from the anterior spinal artery (ASA) on the ventral aspect of the cord can also be seen. (H-J) Orthogonal projections of the inset in E, highlight damage around the base of the dorsal column near the collecting pool of the large dorsal spinal vein.

**Supplementary Fig. 1:**
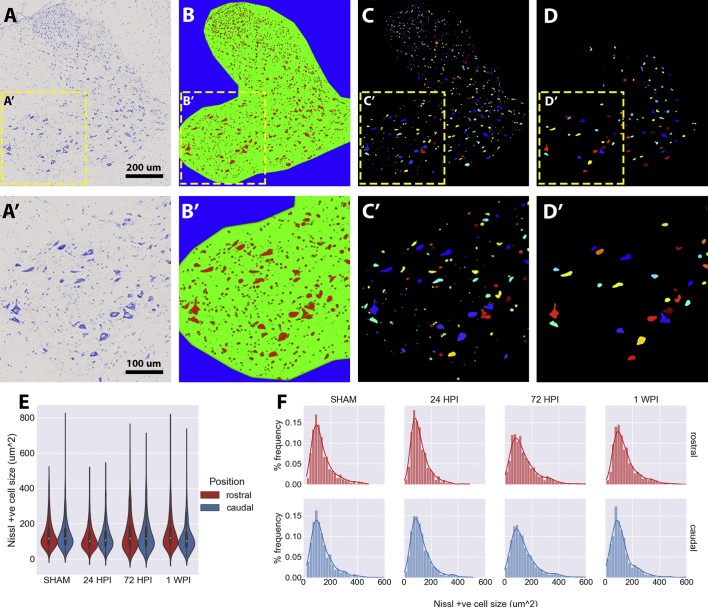
Cell size-frequency distribution of Nissl stained neurons in rostral and caudal spinal cord regions. (A) Representative Nissl stained caudal section cut from one of the SHAM SRuCT imaged injury samples. (B) Ilastik trained pixel classifier probability map of Nissl positive regions in (A). Red = Nissl positive, Green = Gray matter, Blue = Background. (C) CellProfiler segmented Nissl positive objects from (C). (D) Nissl positive neurons identified as those Nissl positive objects in (C), overlapping a manually identified nucleus. (A-D') Insets of A-D. (E) Violin plot of the kernel density estimation of those Nissl positive cells identified in (D), overlayed on a box and whisker plot of data. (F) Cell-size frequency distribution binned to 20 um^2^ bins for those Nissl positive neurons identified in (D). SHAM (*n* = 3), 24 HPI (*n* = 5), 72 HPI (n = 5), and 1 WPI (n = 5). 1 rostral and 1 caudal section were used for quantification, which were matched to those rostral caudal regions as defined in the gray shading of Fig. 4.A.

Acute caudal, but not rostral, swelling in gray matter led us to examine pathology more closely in the border regions adjacent to the injury epicentre (Fig. 6.A-D, Supplementary Fig. 1). Though gray matter swelling at early post-injury time points might be explained by swollen, chromatolytic neuronal cell bodies, we found no significant difference in the cell size distribution of Nissl stained neuronal cell bodies across acute time points (24 HPI, 72 HPI, 1WPI), or between rostral and caudal regions (Supplementary Fig. 1.*E*-F).

Examination of caudal border regions in tomograms at acute time points revealed a high intensity gray level signal local to the base of the dorsal columns, spreading from the epicentre just above the gray matter commissure at 24 HPI (Fig. 6.A, cyan arrows). This signal's location aligned with the emergence of tissue damage at 72 HPI (Fig. 6.B) and cavity appearance by 1 WPI (Fig. 6.C, yellow arrows), that eventually collapsed to leave discrete satellite cavities by 5 WPI (Fig. 6.D, yellow arrows). This led us to pursue the origins of the high intensity gray level signal. The high intensity gray level signal was not readily apparent in a single slice image (Fig. 6.E). However, by generating an average intensity projection of 5 sagittal midline slices (121.6 μm thick), features moving in and out of plane including the extent of the high intensity signal became clear (Fig. 6.F). A minimal intensity projection of this same sagittal slice volume helped to emphasise the local vasculature, including large dorsal spinal veins (DSVs, Fig. 6.F-G, green arrows) and central spinal arteries (CSAs, Fig. 6.F-G, pink arrows). Spread of high intensity gray levels that were associated with damage aligned well with the venous drainage sites of DSVs, particularly when observed in orthogonal planes (Fig. 6.H-J). Given the proximity of damage to venous vasculature sites and that the iodine stain used to enhance contrast can bind to amino residue side chains (Kmiec 2016), we suspected the high intensity signal we associated with damage could be protein rich, vasogenic oedema spreading through tissue. This was tested in subsequent histology.

**Fig. 7:**
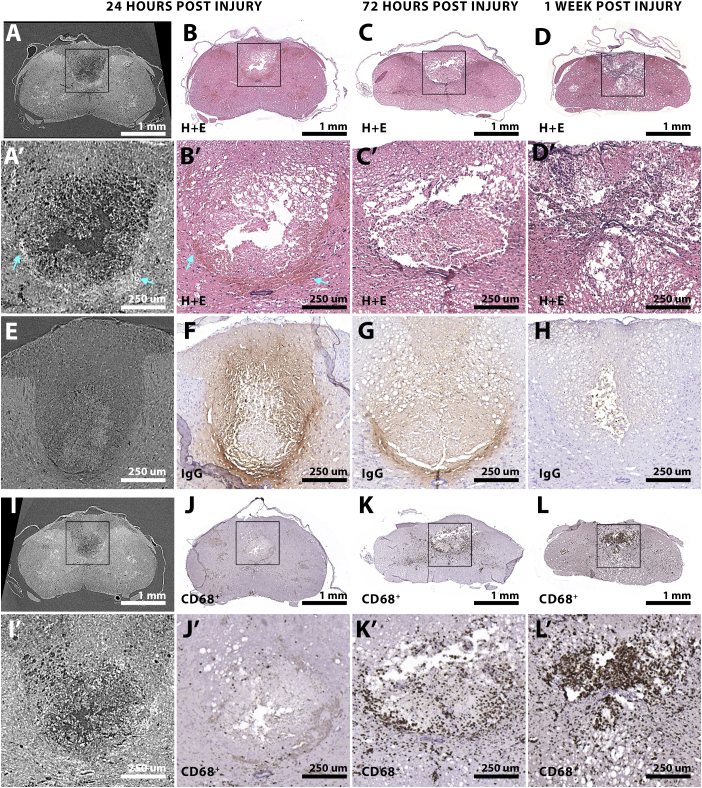
Rostro-caudal tissue border pathology mirrors venous occlusion injury phenotype. (A, A') 6.4 μm thick tomograms of caudal tissue (A' is inset of A) at 24 HPI, aligned to (B, B′) the same region stained with haematoxylin and eosin (H + E). (C, C′) H + E staining at 72 HPI and (D, D') 1 WPI. Haematoxylin (blue) highlights cell nuclei, while eosin (pink) is a protein stain. (E) A 6.4 μm thick tomogram in the caudal region at 24 HPI aligned to (F) IgG staining that marks sites of vasogenic oedema. Though vasogenic oedema subsides by (G) 72 HPI and (H) 1 WPI, it is still most intense at the dorsal column-gray matter interface. (I, I′) 6.4 μm thick tomogram of a caudal region of tissue at 24 HPI aligned to (J, J') the same tissue region at 24 HPI stained for CD68 + ve cells. (K) CD68 + ve cells accumulate by 72 HPI, and by (L) 1 WPI a clear accumulation of CD68 + ve cells within the dorsal column is observed.

Haematoxylin and eosin (H + E) stained tissues aligned to respective tomograms, demonstrated that the high intensity tomogram signal in caudal regions at 24 HPI (Fig. 7.A) co-localised with areas of darker eosin staining (a non-specific protein stain), indicative of oedematous tissue (Fig. 7.B). H + E staining also revealed cells beginning to accumulate within this region at 72 HPI (Fig. 7.C) proceeding to massive cellular accumulation by 1 WPI (Fig. 7.D). As cellular accumulation obscured darker eosin staining at 72 HPI and 1 WPI, we also stained for IgG to reveal oedema regions. This revealed that the high intensity signal in tomograms (Fig. 7.E) did not co-localise with IgG labelling in matched histology at 24 HPI (Fig. 7.F). This lack of overlap was also true at later time points where the high intensity tomogram signal was absent, despite ongoing oedema at the base of the dorsal columns at 72 HPI (Fig. 7.G) that was reduced by 1 WPI (Fig. 7.H). Together, these results dismiss oedema as the source of the high intensity signal in tomograms. The high intensity tomogram signal mainly overlaps regions of tissue debris, suggesting the source of contrast could be ‘edge-enhanced' fine tissue debris. Under the in-line phase contrast imaging conditions used herein, edges of features smaller than 2.82 μm would be artificially bright due to their overlapping Fresnel regimes (Strotton et al. 2018). At 24 HPI, the bright contrast spreading through the base of the dorsal columns, therefore likely highlights a micro-fractured region containing fine tissue debris. This would also explain the high contrast signal surrounding those scattered satellite regions of damage seen in areas far removed from the epicentre (Fig. 2, Fig. 3, cyan arrows).

Finally, to determine the nature of the cellular accumulation in the caudal dorsal columns that was revealed by H + E, CD68 immunostaining was performed. While several CD68 positive cells are present at 24 HPI (Fig. 7.J), their accumulation from 72 HPI (Fig. 7.K) to 1 WPI (Fig. 7.L) reflects the emergence of inflammation in caudal regions at this time point. Notably, the accumulation of CD68 positive cell bodies is limited to a region confined by the same dorsal column - gray matter interface that defines the edge of damage spreading through the base of the dorsal columns.

### 3.5 Behavioural assessments reveal fast sensory and slower motor recovery in the C6 midline contusion model

**Supplementary Fig. 2:**
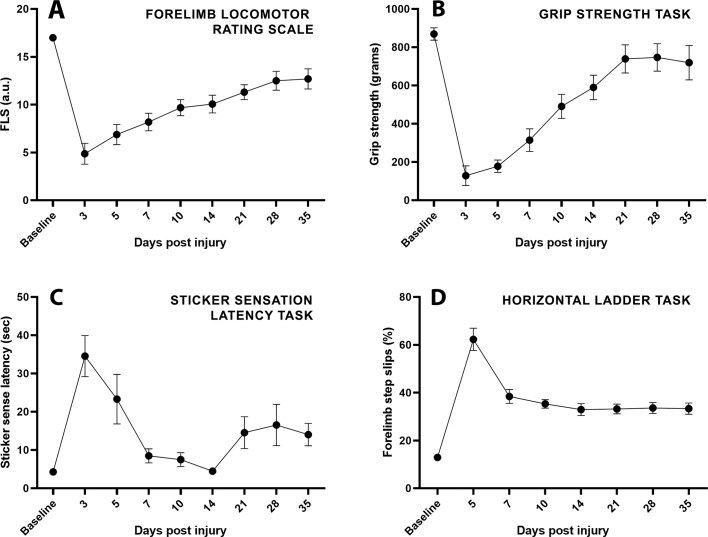
Motor and sensory behavioural recovery following a C6 midline 225 kDyne contusion injury. (A) Forelimb locomotor scale (FLS) (B) Grip strength task (C) Sticker sensation latency task and (D) Forelimb slips on the horizontal ladder task. All graphs are mean ± SEM, *n* = 8.

To give an indication of how the spatiotemporal spread of pathology observed after cervical spinal cord contusion injury may relate to behavioural outcome, multiple behaviour assays for forelimb motor and sensory recovery were conducted on a separate cohort of experimental animals. This cohort received the same C6, 225 kDyne midline contusion injury as animals that were assessed using SRμCT. In these animals, forelimb locomotor score (FLS) assessments showed gradual recovery across the pathology time course which plateaued around 3–5 WPI, from 4.875 ± 1.101 at 72 HPI (between extensive movement of 1–2 joints) to 12.68 ± 1.056 at 5 WPI (forelimb plantar stepping, no toe clearance; Supplementary Fig. 2.A). Grip strength revealed a similar pattern of motor recovery, plateauing to 82.7% of baseline by 3 WPI (Supplementary Fig. 2.B). A forelimb sticker sensation latency task to assess sensory recovery, took 8.01-fold longer at 72 HPI relative to baseline. This rapidly recovered to 1.97-fold longer by 1 WPI, with a slight increased latency after 3 WPI that was attributed to loss of task novelty (Supplementary Fig. 2.C). Forelimb slips as a percentage of steps on the horizontal ladder task (which has both motor and sensory components) showed a similar pattern of sub-acute recovery to the sticker task. Forelimb slips increased 4.81 fold relative to baseline at 5 DPI (earliest time point that could be measured), improving to 2.97 fold by 1 WPI and plateauing to a sustained deficit of ~2.5 fold more slips than baseline beyond 2 WPI (Supplementary Fig. 2.D). Overall, this showed a steady recovery of motor behaviour over the injury time course and a faster, sub-acute recovery of sensory associated behaviours between 72 HPI to 1 WPI. This sensory recovery time course may reflect the loss of acute swelling from 72 HPI to 1 WPI in the caudal dorsal columns (particularly the fasciculus cuneatus) and caudal gray matter.

## 4 Discussion

3D imaging of the intact rat spinal cord by SRμCT generated high-resolution data suitable for multiscale exploration of spinal cord structures and accurate volume measurements, which we subsequently applied to evaluate SCI pathology. Raw volume changes of macro features could be accurately quantified in exactly spaced transverse tissue sections generated *in silico*, between samples aligned to spinal cord root entry zones for time point comparisons. Quantifying these volumes revealed asymmetries in the rostro-caudal spread of pathology, while panning multiple imaging planes and projecting tissue at different thicknesses revealed acute satellite damage foci and fine tissue debris damage spreading along the base of the dorsal columns at acute time points. Such features would be missed with low-resolution 3D imaging and likely overlooked in intermittent 2D histological tissue sections.

Much like tissue-clearing and light sheet microscopy have delivered a third dimension to fluorescent imaging, high-resolution SRμCT, can be used to add a third dimension to histological stains. Indeed, metals found in golgi (Fonseca et al. 2018) and other chemical histological stains (Marina et al. 2019; Strotton et al. 2018) provide region specific contrast to uCT imagery. This comparison highlights the relative merits of light-sheet *vs.* uCT based 3D imaging methods. While light-sheet microscopy can track specific features targeted with fluorescent probes, SRμCT contrast – derived from structural features intrinsic to tissue – delivers unbiased (contrast doesn't depend on label specificity, selectivity or stability) information on tissue content. In our case, contrast arising from tissue structural features was particularly suitable to assess tissue damage and pathological changes that occur at the macroscale following SCI, as well as reveal different white matter tracts in the dorsal column.

SRμCT revealed how following contusion trauma, damaged tissue first breaks down to fine debris before cavity formation. This proceeds rapidly at the injury epicentre, but SRμCT also reveals that tissue damage extends along the base of the dorsal columns into neighbouring tissue segments as early as 24 HPI. The longitudinal spread of pathology through the spinal cord has been observed by MRI clinically, with descriptions of the linear spread of ‘pencil like lesions’ extending through the posterior columns of heterogeneous human SCIs (Ito et al. 1997). Based on *post mortem* observations, Tator (1995) previously described the base of the dorsal columns as a weak haemorrhage point flanking the injury epicentre. Evidence for this region being a weak point for damage spread through the spinal cord is also supported by intraparenchymal injections of a gelatine bolus into rat spinal cord spreading extensively along the cord longitudinal axis (not radially), almost entirely through the base of the dorsal columns (Tomko et al. 2017). This region could therefore be particularly susceptible to damage, due to the physical mismatch of anisotropic white matter and isotropic gray matter at this point (Koser et al. 2015). That this damage is established by 24 HPI and defines the longitudinal extent of tissue damage, suggests much of the extent of a SCI is already determined by primary damage arising from the initial trauma.

While 3D tomograms of whole *ex vivo* tissue are free from 2D histology mechanical sectioning artefacts, artefacts incurred during tissue perfusion and extraction would still be present. However, our *ex vivo* SRμCT findings are comparable with previous *in vivo* MRI measurements of ~25% cord swelling observed at 12 h after a midline contusion injury (Bilgen et al. 2000), and ~ 28% at 8 h after injury (Balentine 1978). High resolution *in vivo* ultrasound has similarly measured an expansion of parenchymal haemorrhage in the cord epicentre by ~19% at 24 HPI, driving tissue swelling to a similar degree as observed here (Soubeyrand et al. 2014). *Ex vivo* SRμCT cord atrophy is also comparable to *in vivo* MRI measurements of 10–17% atrophy at 1 WPI (Deng et al. 2007; Qian et al. 2010). At later time points however, discrepancies emerge between *in vivo* and *ex vivo* volume measurements. *In vivo* DTI atrophy of ~17% at 4 WPI, ~20% at 5 WPI (Patel et al. 2016) and ~ 25% at 8 WPI (Qian et al. 2010), is less than half the near 50% atrophy measured by SRμCT at 5 WPI in the current study. This discrepancy may be due to our use of a midline cervical level contusion injury *versus* thoracic level T12 (Patel et al. 2016) or T7 (Qian et al. 2010) injury. Equally, it may be due to differences in the state of the spinal cord *in vivo vs ex vivo*. Cord extraction may lead to loss of hydrostatic pressure that would support the chronic cavity formed at this time point. Indeed, chronic atrophy measured here at 5 WPI is consistent with spinal cord *ex vivo* atrophy assessments of thoracic cord injury models of ~45% at 8 WPI measured by 2D histology (Chen et al. 2016), and ~ 80% by 8 WPI using *ex vivo* MRI (Scholtes et al. 2011). As tissue collapse is not consistent across time points, normalising internal cord features to cord cross sectional area to compare time points may mask, or reveal false differences between time points. This emphasises the importance of considering raw white and gray matter volume changes across a pathology time course.

Damaged white and gray matter regions align well with damaged vasculature that is also revealed by SRμCT. The precipitous loss of epicentre gray matter following injury is linked to a haemorrhaging local vasculature with little redundancy (Tator and Fehlings 1991). Unlike peripheral white matter (supplied by the centrifugal blood supply), central gray matter is supplied entirely by the centripetal vasculature, formed of central spinal arteries (CSA) that branch from the anterior spinal artery, bifurcating below the central canal to feed the left and right gray matter. As in earlier reports, CSAs cannot be identified within the epicentre at 24 HPI (Cao et al. 2017; Hu et al. 2015). Their absence starves the central gray matter of blood supply. Spared gray matter at the injury epicentre is typically found only in the superficial lamina of the dorsal horn, an area partly vascularised by the peripheral centrifugal system (Thron et al. 1988). While a damaged arterial blood supply contributes to acute gray matter loss, the rostro-caudal spread of dorsal column white matter loss appears linked to sites of damaged venous drainage. At the base of the dorsal columns, venules drain to large, dorsal spinal veins (DSVs). In reduced injury models that perturb venous drainage by ligating these DSVs, extensive ‘oedematous tissue’ forms within the dorsal columns by 24 HPI, and subsides by 72 HPI, before foamy macrophage infiltration at 1 and 2 WPI (Zhang et al. 2001). This is limited to the dorsal columns, almost exactly mirroring dorsal column rostral-caudal pathology following contusion injury. Dorsal spinal vein cauterisation (Martinez-Arizala et al. 1995), or resin blocking of the internal vertebral venous plexus (Zhang et al. (1997) describing Shin (1972)) similarly focuses damage to the dorsal columns. Dorsal white matter is only drained by a peripheral venous plexus, while gray matter is also drained by a central venous system (Griessenauer et al. 2015; Thron et al. 1988). Hence, dorsal white matter may be ‘at risk’ following injury, as there is little redundancy in venous drainage. Venous occlusion models are rarely used in SCI studies, but given their similarities to rostral/caudal damage spread following contusion injury, and the relatively delayed spread of pathology to these regions, they may be an effective reduced injury model in which to study protective treatments targeting these ‘at risk’ regions.

The main rostro-caudal asymmetries that we observed in progressive tissue pathology were faster rostral than caudal white matter loss, and acute gray matter and dorsal column (mostly within the FC) swelling caudal, but not rostral, to the injury epicentre. Acute gray matter and dorsal column swelling (particularly in the FC) was seen caudal, but not rostral to injury. A reduction in caudal swelling by 72 HPI, correlated with the sub-acute (1 WPI) recovery of sensory associated behaviours measured in a second cohort of experimental animals. As the FC conveys forelimb sensory information to the somatosensory cortex (*via* the thalamus), this subsidence of swelling may underlie the rapid sensory recovery.

Finer analysis of dorsal column white matter tracts revealed other asymmetries in pathology progression. Rostral FG volume decline underlies most of the rostral dorsal column atrophy, contrasting rostral dCST and FC tracts, which showed no significant volume changes over our pathology time course. This difference is presumably due to the greater likelihood of the medially located FG being more impacted by the midline contusion injury. In contrast, the lateralised position of FC axons in the dorsal columns makes them more prone to sparing across the injury epicentre (where there is some lateral preservation).

It might be expected that following complete ablation of epicentre dCST, there would be a decline in caudal dCST volume by 5 WPI, but this was not observed. White matter loss can be attributed to both demyelination and Wallerian degeneration of axons. Though not quantified here, previous studies have revealed that rostral dorsal column white matter loss results from both these processes, while caudal degeneration is predominantly due to demyelination (Chen et al. 2016; Ek et al. 2010). This may explain why white matter loss progresses faster in rostral, rather than caudal dorsal columns at 5 WPI. It might also explain why no large changes in caudal dCST volume were seen, as lower levels of demyelination are contributing to volume loss relative to rostral processes. Volume loss in caudal dCST may require a longer time course to observed. White matter loss has been observed at 8 WPI in regions up to 9 mm above and below the injury epicentre (Chen et al. 2016; Ek et al. 2010). Future studies should characterise a longer time course across more extensive spinal cord regions to determine the full extent to which this occurs.

Neither oedema, nor swelling of gray matter neuronal cell bodies seemed to explain the acute swelling seen in caudal gray matter. Another potential source of swelling in caudal gray matter and dorsal columns, may be the loss of descending sympathetic control over basal-tone in the neurovascular unit below, but not above the injury epicentre. This would cause caudal vasculature to dilate freely. The role of descending control over vascular tone has been demonstrated at chronic time points following SCI, where compensatory mechanisms can lead to deleterious, chronic vasoconstriction caudal (though not rostral) to the injury epicentre (Li et al. 2017). Measuring blood flow above and below the injury could test this. While blood flow has previously been reported to uniformly drop rostral and caudal to a SCI epicentre (Rivlin and Tator 1978; Soubeyrand et al. 2013), these reports are restricted to dorsal aspect surface polarography measurements that do not capture the major blood supply from ventral aspect CSAs. Blood flow changes across the full cross sectional area and rostro-caudal spinal axis after SCI are notably absent within experimental SCI literature, but should be obtainable with high temporal and spatial resolution methods such as ultrafast ultrasound (Errico et al. 2015). As the route for systemic drugs to reach the spinal cord, this should be elaborated for potential knock-on effects in terms of how therapeutic treatments target injury.

## 5 Conclusion

In-line phase contrast SRμCT is a rapid 3D imaging technique, suitable for non-destructive screening of large tissue volumes with soft tissue contrast at micrometre resolution. SRμCT enabled raw volume quantification of tissue features across the cervical enlargement of rat spinal cord tissue samples at multiple time points after injury, which could then be aligned to whole cord anatomical features for accurate comparison. This confirmed acute-to-chronic hallmark patterns of progressive tissue pathology development following cervical level spinal cord contusion injury. Rostro-caudal asymmetries in white matter volume changes were also revealed, alongside novel observations of acute swelling of caudal gray and dorsal column white matter after injury. Panning 3D volumes highlighted rostro-caudal damage spread along the base of the dorsal columns at acute time points, which would likely be missed in low-resolution 3D imaging or cleared tissue samples, and not captured in 2D histological slices. The longitudinal extent of tissue damage present at acute time points may limit the efficacy of protective interventions seeking to minimise segmental spread of tissue damage, the extent of which appears well established at acute time points. However, relatively delayed damage to dorsal column white matter associated with sites of venous vasculature could represent a feasible target for limiting pathology spread in a treatable time window. Future investigations using venous injury models that mirror pathology progression in rostro-caudal dorsal columns will be of use in establishing the feasibility of this approach.

The following are the supplementary data related to this article.

Supplementary Video 1 SHAM spinal cord transverse cross-section flythrough. A transverse flythrough of the SHAM rat spinal cord shown in Fig. 1. Imagery is an 8-bit compression of raw data binned with a mean average 2 by 2 by 2 (xyz) filter and flown through at 60 frames per second. Scale and slice depth indicated per frame. Supplementary Video 1

Supplementary Video 2 24 h post spinal cord injury transverse cross section fly through. A transverse fly through of the 24 h post injury rat spinal cord shown in Fig. 2.A. Imagery is an 8-bit compression of raw data binned with a mean average 2 by 2 by 2 (xyz) filter and flown through at 60 frames per second. Scale and slice depth indicated per frame. Supplementary Video 2

Supplementary Video 3 72 h post spinal cord injury transverse cross section fly through. A transverse fly through of the 72 h post injury rat spinal cord shown in Fig. 2.B. Imagery is an 8-bit compression of raw data binned with a mean average 2 by 2 by 2 (xyz) filter and flown through at 60 frames per second. Scale and slice depth indicated per frame. Supplementary Video 3

Supplementary Video 4 1 week post spinal cord injury transverse cross section fly through. A transverse fly through of the 1-week post injury rat spinal cord shown in Fig. 2.C. Imagery is an 8-bit compression of raw data binned with a mean average 2 by 2 by 2 (xyz) filter and flown through at 60 frames per second. Scale and slice depth indicated per frame. Supplementary Video 4

Supplementary Video 5 5 weeks post spinal cord injury transverse cross section fly through. A transverse fly through of the 5-week post injury rat spinal cord shown in Fig. 2.D. Imagery is an 8-bit compression of raw data binned with a mean average 2 by 2 by 2 (xyz) filter and flown through at 60 frames per second. Scale and slice depth indicated per frame. Supplementary Video 5

Supplementary Video 6 Combined acute-to-chronic spinal cord injury time points transverse cross section fly through. A transverse fly through of all acute to chronic time points shown in Fig. 2. Imagery is an 8-bit compression of raw data binned with a mean average 2 by 2 by 2 filter (xyz) and flown through at 60 frames per second. Scale and slice depth indicated per frame with injury time point indicated per spinal cord. Supplementary Video 6

Supplementary material Statistics associated with spinal cord injury feature quantification in Fig. 4. All summary statistics associated with multiple comparisons made in Fig. 4. Image 1

## Funding

This work was supported by the King's Bioscience Institute PhD Programme in Biomedical and Translation Science from Guy's and St Thomas' charity to M.S. and the 10.13039/501100000265Medical Research Council UK (SNCF Award G1002055) to E.J.B. Tomography was performed at the Diamond-Manchester Imaging Branchline I13-2 of 10.13039/100011889Diamond Light Source as part of projects MT14907 and MT18417.

## Authors contributions

M.S. and E.J.B. designed experiments. M.S., A.B. & K.W. performed SRμCT. K.W. scripted code for sequential tomographic scan automation. M.S. performed tomographic reconstruction and analysis. M.S. & C.H. performed tissue processing and histology. M.S. wrote the manuscript with input from A.B. and E.J.B.

## Declaration of Competing Interest

The authors declare no competing financial or non-financial interests.

## References

[bb0005] National Spinal Cord Injury Statistical Center at the University of Alabama (2019). NSCISC Annual Statistical Report - Complete Public Version.

[bb0010] Ahuja C.S., Wilson J.R., Nori S., Kotter M.R.N., Druschel C., Curt A., Fehlings M.G. (2017). Traumatic spinal cord injury. Nat Rev Dis Primers.

[bb0015] Atwood R.C., Bodey A.J., Price S.W., Basham M., Drakopoulos M. (2015). A high-throughput system for high-quality tomographic reconstruction of large datasets at diamond light source. Philos Trans A Math Phys Eng Sci.

[bb0020] Balentine J.D. (1978). Pathology of experimental spinal cord trauma. I. the necrotic lesion as a function of vascular injury. Laboratory Investigation.

[bb0025] Berg S., Kutra D., Kroeger T., Straehle C.N., Kausler B.X., Haubold C., Schiegg M., Ales J., Beier T., Rudy M., Eren K., Cervantes J.I., Xu B., Beuttenmueller F., Wolny A., Zhang C., Koethe U., Hamprecht F.A., Kreshuk A. (2019). ilastik: interactive machine learning for (bio)image analysis. Nat. Methods.

[bb0030] Bilgen M., Abbe R., Liu S.J., Narayana P.A. (2000). Spatial and temporal evolution of hemorrhage in the hyperacute phase of experimental spinal cord injury: in vivo magnetic resonance imaging. Magn. Reson. Med..

[bb0035] Bradbury E.J., Burnside E.R. (2019). Moving beyond the glial scar for spinal cord repair. Nat. Commun..

[bb0040] Bunge R.P., Puckett W.R., Becerra J.L., Marcillo A., Quencer R.M. (1993). Observations on the pathology of human spinal cord injury. A review and classification of 22 new cases with details from a case of chronic cord compression with extensive focal demyelination. Adv. Neurol..

[bb0045] Bunge R.P., Puckett W.R., Hiester E.D. (1997). Observations on the pathology of several types of human spinal cord injury, with emphasis on the astrocyte response to penetrating injuries. Adv. Neurol..

[bb0050] Burnside E.R., De Winter F., Didangelos A., James N.D., Andreica E.C., Layard-Horsfall H., Muir E.M., Verhaagen J., Bradbury E.J. (2018). Immune-evasive gene switch enables regulated delivery of chondroitinase after spinal cord injury. Brain.

[bb0055] Cai R., Pan C., Ghasemigharagoz A., Todorov M.I., Forstera B., Zhao S., Bhatia H.S., Parra-Damas A., Mrowka L., Theodorou D., Rempfler M., Xavier A.L.R., Kress B.T., Benakis C., Steinke H., Liebscher S., Bechmann I., Liesz A., Menze B., Kerschensteiner M., Nedergaard M., Erturk A. (2019). Panoptic imaging of transparent mice reveals whole-body neuronal projections and skull-meninges connections. Nat. Neurosci..

[bb0060] Cao Y., Wu T., Yuan Z., Li D., Ni S., Hu J., Lu H. (2015). Three-dimensional imaging of microvasculature in the rat spinal cord following injury. Sci. Rep..

[bb0065] Cao Y., Zhou Y., Ni S., Wu T., Li P., Liao S., Hu J., Lu H. (2017). Three dimensional quantification of microarchitecture and vessel regeneration by synchrotron radiation microcomputed tomography in a rat model of spinal cord injury. J. Neurotrauma.

[bb0070] Chen K., Liu J., Assinck P., Bhatnagar T., Streijger F., Zhu Q., Dvorak M.F., Kwon B.K., Tetzlaff W., Oxland T.R. (2016). Differential histopathological and behavioral outcomes eight weeks after rat spinal cord injury by contusion, dislocation, and distraction mechanisms. J. Neurotrauma.

[bb0075] Deng X., Ramu J., Narayana P.A. (2007). Spinal cord atrophy in injured rodents: high-resolution MRI. Magn. Reson. Med..

[bb0080] Didangelos A., Puglia M., Iberl M., Sanchez-Bellot C., Roschitzki B., Bradbury E.J. (2016). High-throughput proteomics reveal alarmins as amplifiers of tissue pathology and inflammation after spinal cord injury. Sci. Rep..

[bb0085] Donnelly D.J., Popovich P.G. (2008). Inflammation and its role in neuroprotection, axonal regeneration and functional recovery after spinal cord injury. Exp. Neurol..

[bb0090] Ek C.J., Habgood M.D., Callaway J.K., Dennis R., Dziegielewska K.M., Johansson P.A., Potter A., Wheaton B., Saunders N.R. (2010). Spatio-temporal progression of grey and white matter damage following contusion injury in rat spinal cord. PLoS One.

[bb0095] Ellingson B.M., Kurpad S.N., Schmit B.D. (2008). Ex vivo diffusion tensor imaging and quantitative tractography of the rat spinal cord during long-term recovery from moderate spinal contusion. J. Magn. Reson. Imaging.

[bb0100] Errico C., Pierre J., Pezet S., Desailly Y., Lenkei Z., Couture O., Tanter M. (2015). Ultrafast ultrasound localization microscopy for deep super-resolution vascular imaging. Nature.

[bb0105] Erturk A., Becker K., Jahrling N., Mauch C.P., Hojer C.D., Egen J.G., Hellal F., Bradke F., Sheng M., Dodt H.U. (2012). Three-dimensional imaging of solvent-cleared organs using 3DISCO. Nat. Protoc..

[bb0110] Fonseca M.C., Araujo B.H.S., Dias C.S.B., Archilha N.L., Neto D.P.A., Cavalheiro E., Westfahl H., da Silva A.J.R., Franchini K.G. (2018). High-resolution synchrotron-based X-ray microtomography as a tool to unveil the three-dimensional neuronal architecture of the brain. Sci. Rep..

[bb0115] Gensel J.C., Zhang B. (2015). Macrophage activation and its role in repair and pathology after spinal cord injury. Brain Res..

[bb0120] Greenhalgh A.D., David S., Bennett F.C. (2020). Immune cell regulation of glia during CNS injury and disease. Nat. Rev. Neurosci..

[bb0125] Griessenauer C.J., Raborn J., Foreman P., Shoja M.M., Loukas M., Tubbs R.S. (2015). Venous drainage of the spine and spinal cord: a comprehensive review of its history, embryology, anatomy, physiology, and pathology. Clin. Anat..

[bb0130] Helmchen F., Denk W. (2005). Deep tissue two-photon microscopy. Nat. Methods.

[bb0135] Hu J., Cao Y., Wu T., Li D., Lu H. (2015). 3D angioarchitecture changes after spinal cord injury in rats using synchrotron radiation phase-contrast tomography. Spinal Cord.

[bb0140] Ito T., Oyanagi K., Wakabayashi K., Ikuta F. (1997). Traumatic spinal cord injury: a neuropathological study on the longitudinal spreading of the lesions. Acta Neuropathol..

[bb0145] James N.D., Bartus K., Grist J., Bennett D.L., McMahon S.B., Bradbury E.J. (2011). Conduction failure following spinal cord injury: functional and anatomical changes from acute to chronic stages. J. Neurosci..

[bb0150] James N.D., Shea J., Muir E.M., Verhaagen J., Schneider B.L., Bradbury E.J. (2015). Chondroitinase gene therapy improves upper limb function following cervical contusion injury. Exp. Neurol..

[bb0155] Jirjis M.B., Kurpad S.N., Schmit B.D. (2013). Ex vivo diffusion tensor imaging of spinal cord injury in rats of varying degrees of severity. J. Neurotrauma.

[bb0160] Kak A.C., Slaney M. (2001). Principles of Computerized Tomographic Imaging.

[bb0165] Kmiec Z. (2016). J.a. Kiernan. Histological and histochemical methods: theory and practice. 5th edition, Scion publishing, 2015, 571 pp. Folia Histochem. Cytobiol..

[bb0170] Komotar R.J., Kim G.H., Sughrue M.E., Otten M.L., Rynkowski M.A., Kellner C.P., Hahn D.K., Merkow M.B., Garrett M.C., Starke R.M., Connolly E.S. (2007). Neurologic assessment of somatosensory dysfunction following an experimental rodent model of cerebral ischemia. Nat. Protoc..

[bb0175] Koser D.E., Moeendarbary E., Hanne J., Kuerten S., Franze K. (2015). CNS cell distribution and axon orientation determine local spinal cord mechanical properties. Biophys. J..

[bb0180] Kremer J.R., Mastronarde D.N., McIntosh J.R. (1996). Computer visualization of three-dimensional image data using IMOD. J. Struct. Biol..

[bb0185] Kwon B.K., Okon E., Hillyer J., Mann C., Baptiste D., Weaver L.C., Fehlings M.G., Tetzlaff W. (2011). A systematic review of non-invasive pharmacologic neuroprotective treatments for acute spinal cord injury. J. Neurotrauma.

[bb0190] Li Y., Lucas-Osma A.M., Black S., Bandet M.V., Stephens M.J., Vavrek R., Sanelli L., Fenrich K.K., Di Narzo A.F., Dracheva S., Winship I.R., Fouad K., Bennett D.J. (2017). Pericytes impair capillary blood flow and motor function after chronic spinal cord injury. Nat. Med..

[bb0195] Marina E., Mareike T., Torben R., Wiebke M., Tim S. (2019). Evaluation of different heavy-metal stains and embedding media for phase contrast tomography of neuronal tissue. Proc.SPIE.

[bb0200] Martinez-Arizala A., Mora R.J., Madsen P.W., Green B.A., Hayashi N. (1995). Dorsal spinal venous occlusion in the rat. J. Neurotrauma.

[bb0205] McQuin C., Goodman A., Chernyshev V., Kamentsky L., Cimini B.A., Karhohs K.W., Doan M., Ding L., Rafelski S.M., Thirstrup D., Wiegraebe W., Singh S., Becker T., Caicedo J.C., Carpenter A.E. (2018). CellProfiler 3.0: Next-generation image processing for biology. PLoS Biol.

[bb0210] Metscher B.D. (2009). MicroCT for developmental biology: a versatile tool for high-contrast 3D imaging at histological resolutions. Dev. Dyn..

[bb0215] Metz G.A., Whishaw I.Q. (2009). The ladder rung walking task: a scoring system and its practical application. J Vis Exp..

[bb0220] Meyer O.A., Tilson H.A., Byrd W.C., Riley M.T. (1979). A method for the routine assessment of fore- and hindlimb grip strength of rats and mice. Neurobehav. Toxicol..

[bb0225] Mizutani R., Suzuki Y. (2012). X-ray microtomography in biology. Micron.

[bb0230] Moeendarbary E., Weber I.P., Sheridan G.K., Koser D.E., Soleman S., Haenzi B., Bradbury E.J., Fawcett J., Franze K. (2017). The soft mechanical signature of glial scars in the central nervous system. Nat. Commun..

[bb0235] Noble L.J., Wrathall J.R. (1985). Spinal cord contusion in the rat: morphometric analyses of alterations in the spinal cord. Exp. Neurol..

[bb0240] Norenberg M.D., Smith J., Marcillo A. (2004). The pathology of human spinal cord injury: defining the problems. J. Neurotrauma.

[bb0245] Ouzounov D.G., Wang T., Wang M., Feng D.D., Horton N.G., Cruz-Hernandez J.C., Cheng Y.T., Reimer J., Tolias A.S., Nishimura N., Xu C. (2017). In vivo three-photon imaging of activity of GCaMP6-labeled neurons deep in intact mouse brain. Nat. Methods.

[bb0250] Paganin D., Mayo S.C., Gureyev T.E., Miller P.R., Wilkins S.W. (2002). Simultaneous phase and amplitude extraction from a single defocused image of a homogeneous object. J. Microsc..

[bb0255] Pan C., Cai R., Quacquarelli F.P., Ghasemigharagoz A., Lourbopoulos A., Matryba P., Plesnila N., Dichgans M., Hellal F., Erturk A. (2016). Shrinkage-mediated imaging of entire organs and organisms using uDISCO. Nat. Methods.

[bb0260] Patel S.P., Smith T.D., VanRooyen J.L., Powell D., Cox D.H., Sullivan P.G., Rabchevsky A.G. (2016). Serial diffusion tensor imaging in vivo predicts long-term functional recovery and histopathology in rats following different severities of spinal cord injury. J. Neurotrauma.

[bb0265] Pešić Z., De Fanis A., Wagner U., Rau C. (2013). Experimental stations at I13 beamline at diamond light source. J. Phys. Conf. Ser..

[bb0270] Qian J., Herrera J.J., Narayana P.A. (2010). Neuronal and axonal degeneration in experimental spinal cord injury: in vivo proton magnetic resonance spectroscopy and histology. J. Neurotrauma.

[bb0275] Rau C., Wagner U., Pešić Z., De Fanis A. (2011). Coherent imaging at the diamond beamline I13. Physica status solidi (a).

[bb0280] Richardson D.S., Lichtman J.W. (2015). Clarifying tissue clearing. Cell.

[bb0285] Rivlin A.S., Tator C.H. (1978). Regional spinal cord blood flow in rats after severe cord trauma. J. Neurosurg..

[bb0290] Schneider C.A., Rasband W.S., Eliceiri K.W. (2012). NIH image to ImageJ: 25 years of image analysis. Nat. Methods.

[bb0295] Scholtes F., Theunissen E., Phan-Ba R., Adriaensens P., Brook G., Franzen R., Gelan J., Schoenen J., Martin D. (2011). Post-mortem assessment of rat spinal cord injury and white matter sparing using inversion recovery-supported proton density magnetic resonance imaging. Spinal Cord.

[bb0300] Shin M. (1972). An experimental study of cervical myelopathies due to venous congestion (in Japanese). J. Jpn. Orthop. Assoc..

[bb0305] Singh A., Krisa L., Frederick K.L., Sandrow-Feinberg H., Balasubramanian S., Stackhouse S.K., Murray M., Shumsky J.S. (2014). Forelimb locomotor rating scale for behavioral assessment of recovery after unilateral cervical spinal cord injury in rats. J. Neurosci. Methods.

[bb0310] Soubeyrand M., Laemmel E., Court C., Dubory A., Vicaut E., Duranteau J. (2013). Rat model of spinal cord injury preserving dura mater integrity and allowing measurements of cerebrospinal fluid pressure and spinal cord blood flow. Eur. Spine J..

[bb0315] Soubeyrand M., Badner A., Vawda R., Chung Y.S., Fehlings M.G. (2014). Very high resolution ultrasound imaging for real-time quantitative visualization of vascular disruption after spinal cord injury. J. Neurotrauma.

[bb0320] Strotton M.C., Bodey A.J., Wanelik K., Darrow M.C., Medina E., Hobbs C., Rau C., Bradbury E.J. (2018). Optimising complementary soft tissue synchrotron X-ray microtomography for reversibly-stained central nervous system samples. Sci. Rep..

[bb0325] Tator C.H. (1995). Update on the pathophysiology and pathology of acute spinal cord injury. Brain Pathol..

[bb0330] Tator C.H., Fehlings M.G. (1991). Review of the secondary injury theory of acute spinal cord trauma with emphasis on vascular mechanisms. J. Neurosurg..

[bb0335] Thron A.K., Rossberg C., Mironov A. (1988). Vascular Anatomy of the Spinal Cord : Neuroradiological Investigations and Clinical Syndromes.

[bb0340] Titarenko V. (2016). Analytical formula for two-dimensional ring artefact suppression. J. Synchrotron Radiat..

[bb0345] Titarenko V., Bradley R., Martin C., Withers P.J., Titarenko S. (2010). Regularization methods for inverse problems in x-ray tomography. SPIE Optical Engineering + Applications.

[bb0350] Tomko P., Farkas D., Cizkova D., Vanicky I. (2017). Longitudinal enlargement of the lesion after spinal cord injury in the rat: a consequence of malignant edema?. Spinal Cord.

[bb0355] Wadeson N., Basham M. (2016). Savu: a Python-based, MPI framework for simultaneous processing of multiple, N-dimensional, large tomography datasets.

[bb0360] Zhang Z.-A., Nonaka H., Hatori T. (1997). The microvasculature of the spinal cord in the human adult. Neuropathology.

[bb0365] Zhang Z., Nonaka H., Nagayama T., Hatori T., Ihara F., Zhang L., Akima M. (2001). Circulatory disturbance of rat spinal cord induced by occluding ligation of the dorsal spinal vein. Acta Neuropathol..

