# Supplementary material for: The spatiotemporal spread of cervical spinal cord contusion injury pathology revealed by 3D in-line phase contrast synchrotron X-ray microtomography
Source: Exp Neurol. 2021 Feb;336:113529. doi: 10.1016/j.expneurol.2020.113529 (PMC7840595; doi:10.1016/j.expneurol.2020.113529)
Supplement: Supplementary file 7 — Supplementary material Statistics associated with spinal cord injury feature quantification in Fig. 4. All summary statistics associated with multiple comparisons made in Fig. 4. [file mmc9.pdf]

Additional file 7. Statistics associated with spinal cord injury feature quantification in Fig.4 and Fig.5.

The spatiotemporal spread of spinal cord contusion injury pathology revealed by 3D in-line phase contrast synchrotron X-ray microtomography

Merrick C. Strotton (merrick.strotton@kcl.ac.uk)[1,†,+], Andrew J. Bodey (andrew.bodey@diamond.ac.uk)[2], Kazimir Wanelik (kaz.wanelik@diamond.ac.uk)[2], Carl Hobbs (carl.hobbs@kcl.ac.uk)[1], Christoph Rau (christoph.rau@diamond.ac.uk)[2], Elizabeth J. Bradbury (elizabeth.bradbury@kcl.ac.uk)[1,+].

[1]King's College London, Wolfson Centre for Age Related Diseases, Institute of Psychiatry, Psychology & Neuroscience, Guy's Campus, London Bridge, London, SE1 1UL, U.K.

[2]Diamond Light Source, Oxfordshire, OX11 0DE, UK.

[†]Current affiliation: University of Zurich, Department of Quantitative Biomedicine, Zurich, 8006, Switzerland.

[+]Corresponding authors.

Email: elizabeth.bradbury@kcl.ac.uk, merrick.strotton@uzh.ch

Telephone: +44 (0) 207 848 6183

Supplementary Table.S1.A. Spinal Cord Area, Two-way RM-ANOVA. Relates to Fig.4.A.

ANOVA table	sum-of-squares	DF	mean-squares	F (DFn, DFd)	P value
**Interaction**	235.4764	84	2.80329	F (84, 462) = 13.65565	P<0.0001
**Rostro-caudal distance**	410.239	21	19.53519	F (21, 462) = 95.16163	P<0.0001
**Time point**	811.1625	4	202.7906	F (4, 22) = 39.64291	P<0.0001
**Spinal Cords**	112.5395	22	5.115433	F (22, 462) = 24.91877	P<0.0001
**Residual**	94.84135	462	0.2052843		

		SHAM	24 HPI	72 HPI	1 WPI	5 WPI
**SHAM**	mean difference	0	-0.5433	0.0212	1.119	2.629
	p-value	1	0.0245	>0.999	<0.0001	<0.0001
**24 HPI**	mean difference		0	-0.5450	-1.663	-3.172
	p-value		1	0.0051	<0.0001	<0.0001
**72 HPI**	mean difference			0	-1.098	-2.607
	p-value			1	<0.0001	<0.0001
**1 WPI**	mean difference				0	-1.509
	p-value				1	<0.0001
**5 WPI**	mean difference					0
	p-value					1

Supplementary Table.S1.B. Rostral Spinal Cord Area, One-way ANOVA. Relates to Fig.4.B.

ANOVA table	sum-of-squares	DF	mean-squares	F (DFn, DFd)	P value
**Time point**	111	4	27.9	F (4, 22) = 34.7	P<0.0001
**Spinal Cord Replicates**	17.7	22	0.803		
**Total**	129	26			

		SHAM	24 HPI	72 HPI	1 WPI	5 WPI
**SHAM**	mean difference	0	0.03329	0.3163	1.287	2.233
	p-value	1	>0.9999	0.8113	0.0018	<0.0001
**24 HPI**	mean difference		0	0.2830	1.254	2.2
	p-value		1	0.7564	0.0002	<0.0001
**72 HPI**	mean difference			0	0.9708	1.917
	p-value			1	0.004	<0.0001
**1 WPI**	mean difference				0	0.9459
	p-value				1	0.0052
**5 WPI**	mean difference					0
	p-value					1

Supplementary Table.S1.C. Epicentre Spinal Cord Area, One-way ANOVA. Relates to Fig.4.C.

ANOVA table	sum-of-squares	DF	mean-squares	F (DFn, DFd)	P value
**Time point**	111	4	27.9	F (4, 22) = 34.7	P<0.0001
**Spinal Cord Replicates**	17.7	22	0.803		
**Total**	129	26			

		SHAM	24 HPI	72 HPI	1 WPI	5 WPI
**SHAM**	mean difference	0	-1.71	-0.496	1.35	3.97
	p-value	1	0.0868	0.933	0.2456	<0.0001
**24 HPI**	mean difference		0	1.2100	3.06	5.68
	p-value		1	0.1687	<0.0001	<0.0001
**72 HPI**	mean difference			0	1.84	4.46
	p-value			1	0.0136	<0.0001
**1 WPI**	mean difference				0	2.62
	p-value				1	0.0004
**5 WPI**	mean difference					0
	p-value					1

Supplementary Table.S1.D. Caudal Spinal Cord Area, One-way ANOVA. Relates to Fig.4.D.

ANOVA table	sum-of-squares	DF	mean-squares	F (DFn, DFd)	P value
**Time point**	7.578	4	1.895	F (4, 22) = 27.36	P<0.0001
**Spinal Cord Replicates**	1.523	22	0.06923		
**Total**	9.101	26			

		SHAM	24 HPI	72 HPI	1 WPI	5 WPI
**SHAM**	mean difference	0	-0.3311	-0.1361	0.4463	1.091
	p-value	1	0.4097	0.9468	0.153	<0.0001
**24 HPI**	mean difference		0	0.7034	0.0003	1.422
	p-value		1	0.1687	<0.0001	<0.0001
**72 HPI**	mean difference			0	0.0073	<0.0001
	p-value			1	0.0136	<0.0001
**1 WPI**	mean difference				0	0.0028
	p-value				1	0.0004
**5 WPI**	mean difference					0
	p-value					1

Supplementary Table.S1.E. Spinal Cord Tissue Damage Area, Two-way RM-ANOVA. Relates to Fig.4.E.

ANOVA table	sum-of-squares	DF	mean-squares	F (DFn, DFd)	P value
**Interaction**	90.85	63	1.442	F (63, 420) = 5.597	P<0.0001
**Rostro-caudal distance**	931	21	44.33	F (21, 420) = 172.1	P<0.0001
**Time point**	65.9	3	21.97	F (3, 20) = 14.72	P<0.0001
**Spinal Cords**	29.84	20	1.492	F (20, 420) = 5.791	P<0.0001
**Residual**	108.2	420	0.2576		

		24 HPI	72 HPI	1 WPI	5 WPI
**24 HPI**	mean difference	0	0.5742	-0.0253	-0.4163
	p-value	1	0.0403	0.9990	<0.0001
**72 HPI**	mean difference		0	-0.5995	-0.9905
	p-value		1	0.0229	<0.0001
**1 WPI**	mean difference			0	-0.3910
	p-value			1	0.0359
**5 WPI**	mean difference				0
	p-value				1

Supplementary Table.S1.F. Rostral Spinal Cord Tissue Damage Area, One-way ANOVA. Relates to Fig.4.F.

ANOVA table	sum-of-squares	DF	mean-squares	F (DFn, DFd)	P value
**Time point**	0.126	3	0.04199	F (3, 20) = 7.258	P=0.0018
**Spinal Cord Replicates**	0.1157	20	0.005785		
**Total**	0.2417	23			

		24 HPI	72 HPI	1 WPI	5 WPI
**24 HPI**	mean difference	0	-0.132	-0.0965	0.04859
	p-value	1	0.0326	0.1582	0.6898
**72 HPI**	mean difference		0	0.03547	0.1805
	p-value		1	0.85	0.0028
**1 WPI**	mean difference			0	0.1451
	p-value			1	0.0172
**5 WPI**	mean difference				0
	p-value				1

Supplementary Table.S1.G. Epicentre Spinal Cord Tissue Damage Area, One-way ANOVA. Relates to Fig.4.G.

ANOVA table	sum-of-squares	DF	mean-squares	F (DFn, DFd)	P value
**Time point**	16.56	3	5.521	F (3, 20) = 10.21	P=0.0003
**Spinal Cord Replicates**	10.82	20	0.5409		
**Total**	27.38	23			

		24 HPI	72 HPI	1 WPI	5 WPI
**24 HPI**	mean difference	0	-0.9946	0.2924	1.336
	p-value	1	0.1218	0.9002	0.0241
**72 HPI**	mean difference		0	1.287	2.331
	p-value		1	0.0309	0.0001
**1 WPI**	mean difference			0	1.0440
	p-value			1	0.0979
**5 WPI**	mean difference				0
	p-value				1

Supplementary Table.S1.H. Caudal Spinal Cord Tissue Damage Area, One-way ANOVA. Relates to Fig.4.H.

ANOVA table	sum-of-squares	DF	mean-squares	F (DFn, DFd)	P value
**Time point**	0.04139	3	0.0138	F (3, 20) = 4.677	P=0.0124
**Spinal Cord Replicates**	0.05899	20	0.00295		
**Total**	0.1004	23			

		24 HPI	72 HPI	1 WPI	5 WPI
**24 HPI**	mean difference	0	-0.03688	0.0273	0.077
	p-value	1	0.6483	0.8192	0.0985
**72 HPI**	mean difference		0	0.06422	0.1139
	p-value		1	0.2043	0.0083
**1 WPI**	mean difference			0	0.0497
	p-value			1	0.4098
**5 WPI**	mean difference				0
	p-value				1

Supplementary Table.S1.I. Spinal Cord Gray Matter Area, Two-way RM-ANOVA. Relates to Fig.4.I.

ANOVA table	sum-of-squares	DF	mean-squares	F (DFn, DFd)	P value
Interaction	58.31061	84	0.694174	F (84, 462) = 9.125212	P<0.0001
Rostro-caudal distance	107.6171	21	5.124622	F (21, 462) = 67.36533	P<0.0001
Time point	121.2405	4	30.31013	F (4, 22) = 49.44984	P<0.0001
Spinal Cords	13.48483	22	0.6129468	F (22, 462) = 8.057447	P<0.0001
Residual	35.14531	462	0.07607209		

		SHAM	24 HPI	72 HPI	1 WPI	5 WPI
SHAM	mean difference	0	-0.2025	0.2877	0.6638	1.018
	p-value	1	0.0526	0.0107	<0.0001	<0.0001
24 HPI	mean difference		0	-0.4902	-0.8863	-3.172
	p-value		1	<0.0001	<0.0001	<0.0001
72 HPI	mean difference			0	-0.3961	-0.7307
	p-value			1	<0.0001	<0.0001
1 WPI	mean difference				0	-0.3346
	p-value				1	0.0007
5 WPI	mean difference					0
	p-value					1

Supplementary Table.S1.J. Rostral Spinal Cord Gray Matter Area, One-way ANOVA. Relates to Fig.4.J.

ANOVA table	sum-of-squares	DF	mean-squares	F (DFn, DFd)	P value
Time point	1.998	4	0.4996	F (4, 22) = 22.83	P<0.0001
Spinal Cord Replicates	0.4815	22	0.02189		
Total	2.48	26			

		SHAM	24 HPI	72 HPI	1 WPI	5 WPI
SHAM	mean difference	0	-0.01991	-0.0208	0.4659	0.5846
	p-value	1	0.9997	0.9996	0.0017	0.0001
24 HPI	mean difference		0	-0.0009	0.4858	0.6045
	p-value		1	>0.9999	<0.0001	<0.0001
72 HPI	mean difference			0	0.4867	0.6054
	p-value			1	<0.0001	<0.0001
1 WPI	mean difference				0	0.1187
	p-value				1	0.6404
5 WPI	mean difference					0
	p-value					1

Supplementary Table.S1.K. Epicentre Spinal Cord Gray Matter Area, One-way ANOVA. Relates to Fig.4.K.

ANOVA table	sum-of-squares	DF	mean-squares	F (DFn, DFd)	P value
Time point	21.26	4	5.314	F (4, 22) = 36.70	P<0.0001
Spinal Cord Replicates	3.186	22	0.1448		
Total	24.44	26			

		SHAM	24 HPI	72 HPI	1 WPI	5 WPI
SHAM	mean difference	0	-0.1663	1.133	1.464	2.205
	p-value	1	0.9706	0.003	0.0002	<0.0001
24 HPI	mean difference		0	1.3000	1.631	2.372
	p-value		1	<0.0001	<0.0001	<0.0001
72 HPI	mean difference			0	0.3308	1.072
	p-value			1	0.5697	0.0006
1 WPI	mean difference				0	0.7411
	p-value				1	0.0207
5 WPI	mean difference					0
	p-value					1

Supplementary Table.S1.L. Caudal Spinal Cord Gray Matter Area, One-way ANOVA. Relates to Fig.4.L.

ANOVA table	sum-of-squares	DF	mean-squares	F (DFn, DFd)	P value
Time point	1.377	4	0.3441	F (4, 22) = 28.39	P<0.0001
Spinal Cord Replicates	0.2667	22	0.01212		
Total	1.643	26			

		SHAM	24 HPI	72 HPI	1 WPI	5 WPI
SHAM	mean difference	0	-0.3207	-0.2856	0.1299	0.2096
	p-value	1	0.0037	0.0106	0.4724	0.0877
24 HPI	mean difference		0	0.0351	0.4507	0.5303
	p-value		1	0.9805	<0.0001	<0.0001
72 HPI	mean difference			0	0.4156	0.4952
	p-value			1	<0.0001	<0.0001
1 WPI	mean difference				0	0.07964
	p-value				1	0.7214
5 WPI	mean difference					0
	p-value					1

Supplementary Table.S1.M. Spinal Cord White Matter Area, Two-way RM-ANOVA. Relates to Fig.4.M.

ANOVA table	sum-of-squares	DF	mean-squares	F (DFn, DFd)	P value
Interaction	81.30873	84	0.9679611	F (84, 462) = 7.791287	P<0.0001
Rostro-caudal distance	436.8995	21	20.80474	F (21, 462) = 167.4610	P<0.0001
Time point	262.3562	4	65.58906	F (4, 22) = 35.96413	P<0.0001
Spinal Cords	40.12218	22	1.823735	F (22, 462) = 14.67956	P<0.0001
Residual	57.39719	462	0.1242364		

		SHAM	24 HPI	72 HPI	1 WPI	5 WPI
SHAM	mean difference	0	0.618	1.345	1.447	2.122
	p-value	1	<0.0001	<0.0001	<0.0001	<0.0001
24 HPI	mean difference		0	-0.7270	-0.8294	-1.504
	p-value		1	<0.0001	<0.0001	<0.0001
72 HPI	mean difference			0	-0.1025	-0.7773
	p-value			1	0.9561	<0.0001
1 WPI	mean difference				0	-0.6748
	p-value				1	<0.0001
5 WPI	mean difference					0
	p-value					1

Supplementary Table.S1.N. Rostral Spinal Cord White Matter Area, One-way ANOVA. Relates to Fig.4.N.

ANOVA table	sum-of-squares	DF	mean-squares	F (DFn, DFd)	P value
Time point	7.132	4	1.783	F (4, 22) = 16.06	P<0.0001
Spinal Cord Replicates	2.442	22	0.111		
Total	9.575	26			

		SHAM	24 HPI	72 HPI	1 WPI	5 WPI
SHAM	mean difference	0	0.1969	0.5381	0.9866	1.463
	p-value	1	0.9166	0.1877	0.0032	<0.0001
24 HPI	mean difference		0	0.3412	0.7898	1.266
	p-value		1	0.4129	0.0038	<0.0001
72 HPI	mean difference			0	0.4486	0.9251
	p-value			1	0.1724	0.0007
1 WPI	mean difference				0	0.4765
	p-value				1	0.1324
5 WPI	mean difference					0
	p-value					1

Supplementary Table.S1.O. Epicentre Spinal Cord White Matter Area, One-way ANOVA. Relates to Fig.4.O.

ANOVA table	sum-of-squares	DF	mean-squares	F (DFn, DFd)	P value
Time point	36.29	4	9.074	F (4, 22) = 56.55	P<0.0001
Spinal Cord Replicates	3.53	22	0.1605		
Total	39.82	26			

		SHAM	24 HPI	72 HPI	1 WPI	5 WPI
SHAM	mean difference	0	0.1969	0.5381	0.9866	1.463
	p-value	1	0.9166	0.1877	0.0032	<0.0001
24 HPI	mean difference		0	0.3412	0.7898	1.266
	p-value		1	0.4129	0.0038	<0.0001
72 HPI	mean difference			0	0.4486	0.9251
	p-value			1	0.1724	0.0007
1 WPI	mean difference				0	0.4765
	p-value				1	0.1324
5 WPI	mean difference					0
	p-value					1

Supplementary Table.S1.P. Caudal Spinal Cord White Matter Area, One-way ANOVA. Relates to Fig.4.P.

ANOVA table	sum-of-squares	DF	mean-squares	F (DFn, DFd)	P value
Time point	2.808	4	0.7019	F (4, 22) = 13.77	P<0.0001
Spinal Cord Replicates	1.122	22	0.05098		
Total	3.929	26			

		SHAM	24 HPI	72 HPI	1 WPI	5 WPI
SHAM	mean difference	0	-0.02746	0.2809	0.3835	0.8596
	p-value	1	0.9998	0.4207	0.1521	0.0002
24 HPI	mean difference		0	0.3084	0.4109	0.887
	p-value		1	0.1624	0.0337	<0.0001
72 HPI	mean difference			0	0.1026	0.5787
	p-value			1	0.9318	0.0017
1 WPI	mean difference				0	0.4761
	p-value				1	0.011
5 WPI	mean difference					0
	p-value					1

Supplementary Table.S1.Q. Spinal Cord Dorsal Column Area, Two-way RM-ANOVA. Relates to Fig.4.Q.

ANOVA table	sum-of-squares	DF	mean-squares	F (DFn, DFd)	P value
Interaction	12.14648	84	0.1446009	F (84, 462) = 7.198088	P<0.0001
Rostro-caudal distance	65.25181	21	3.107229	F (21, 462) = 154.6747	P<0.0001
Time point	7.022556	4	1.755639	F (4, 22) = 14.13156	P<0.0001
Spinal Cords	2.733177	22	0.1242353	F (22, 462) = 6.184309	P<0.0001
Residual	9.281024	462	0.0200888		

		SHAM	24 HPI	72 HPI	1 WPI	5 WPI
SHAM	mean difference	0	0.1031	0.2443	0.2516	0.3397
	p-value	1	0.1922	<0.0001	<0.0001	<0.0001
24 HPI	mean difference		0	-0.7270	-0.8294	-1.504
	p-value		1	<0.0001	<0.0001	<0.0001
72 HPI	mean difference			0	-0.1025	-0.7773
	p-value			1	0.9561	<0.0001
1 WPI	mean difference				0	-0.0881
	p-value				1	0.4092
5 WPI	mean difference					0
	p-value					1

Supplementary Table.S1.R. Rostral Spinal Cord Dorsal Column Area, One-way ANOVA. Relates to Fig.4.R.

ANOVA table	sum-of-squares	DF	mean-squares	F (DFn, DFd)	P value
Time point	0.4343	4	0.1086	F (4, 22) = 11.37	P<0.0001
Spinal Cord Replicates	0.21	22	0.009547		
Total	0.6443	26			

		SHAM	24 HPI	72 HPI	1 WPI	5 WPI
SHAM	mean difference	0	-0.0933	-0.1483	0.0474	0.1986
	p-value	1	0.664	0.2365	0.9574	0.0607
24 HPI	mean difference		0	-0.0550	0.1407	0.2919
	p-value		1	0.8632	0.1283	0.0003
72 HPI	mean difference			0	0.1957	0.3469
	p-value			1	0.0167	<0.0001
1 WPI	mean difference				0	0.1512
	p-value				1	0.0898
5 WPI	mean difference					0
	p-value					1

Supplementary Table.S1.S. Epicentre Spinal Cord Dorsal Column Area, One-way ANOVA. Relates to Fig.4.S.

ANOVA table	sum-of-squares	DF	mean-squares	F (DFn, DFd)	P value
Time point	1.72	4	0.43	F (4, 22) = 31.64	P<0.0001
Spinal Cord Replicates	0.299	22	0.01359		
Total	2.019	26			

		SHAM	24 HPI	72 HPI	1 WPI	5 WPI
SHAM	mean difference	0	0.5952	0.8186	0.8359	0.7537
	p-value	1	<0.0001	<0.0001	<0.0001	<0.0001
24 HPI	mean difference		0	0.2234	0.2407	0.1585
	p-value		1	0.0233	0.0131	0.1655
72 HPI	mean difference			0	0.01727	-0.06493
	p-value			1	0.999	0.8679
1 WPI	mean difference				0	-0.0822
	p-value				1	0.7394
5 WPI	mean difference					0
	p-value					1

Supplementary Table.S1.T. Caudal Spinal Cord Dorsal Column Area, One-way ANOVA. Relates to Fig.4.T.

ANOVA table	sum-of-squares	DF	mean-squares	F (DFn, DFd)	P value
Time point	0.3389	4	0.08472	F (4, 22) = 17.99	P<0.0001
Spinal Cord Replicates	0.1036	22	0.004709		
Total	0.4425	26			

		SHAM	24 HPI	72 HPI	1 WPI	5 WPI
SHAM	mean difference	0	-0.2297	-0.262	-0.106	0.01178
	p-value	1	0.0009	0.0002	0.2222	0.9992
24 HPI	mean difference		0	-0.0323	0.1237	0.2415
	p-value		1	0.9232	0.036	<0.0001
72 HPI	mean difference			0	0.156	0.2738
	p-value			1	0.0057	<0.0001
1 WPI	mean difference				0	0.1178
	p-value				1	0.0494
5 WPI	mean difference					0
	p-value					1

Supplementary Table.S2.A. Dorsal corticospinal tract Area, Two-way RM-ANOVA. Relates to Fig.5.A.

ANOVA table	sum-of-squares	DF	mean-squares	F (DFn, DFd)	P value
Interaction	0.8942242	84	0.01064553	F (84, 462) = 4.805635	P<0.0001
Rostro-caudal distance	2.399295	21	0.1142522	F (21, 462) = 51.57605	P<0.0001
Time point	0.5264902	4	0.1316226	F (4, 22) = 20.61509	P<0.0001
Spinal Cords	0.1404648	22	0.006384766	F (22, 462) = 2.882230	P<0.0001
Residual	1.02343	462	0.002215217		

		SHAM	24 HPI	72 HPI	1 WPI	5 WPI
SHAM	mean difference	0	0.0664	0.0812	0.0954	0.1006
	p-value	1	0.0001	<0.0001	<0.0001	<0.0001
24 HPI	mean difference		0	-0.0148	-0.0290	-0.0342
	p-value		1	0.5691	0.0518	0.0165
72 HPI	mean difference			0	-0.0142	-0.0193
	p-value			1	0.6074	0.3141
1 WPI	mean difference				0	-0.0051
	p-value				1	0.9841
5 WPI	mean difference					0
	p-value					1

Supplementary Table.S2.B. Rostral Dorsal corticospinal tract Area, One-way ANOVA. Relates to Fig.5.B.

ANOVA table	sum-of-squares	DF	mean-squares	F (DFn, DFd)	P value
Time point	0.03685	4	0.009212	F (4, 22) = 4.150	P=0.0118
Spinal Cord Replicates	0.04883	22	0.00222		
Total	0.08568	26			

		SHAM	24 HPI	72 HPI	1 WPI	5 WPI
SHAM	mean difference	0	0.01719	0.02686	-0.04676	-0.06225
	p-value	1	0.9848	0.9259	0.6319	0.3624
24 HPI	mean difference		0	0.0097	-0.06395	-0.07944
	p-value		1	0.9963	0.1667	0.0551
72 HPI	mean difference			0	-0.07362	-0.08912
	p-value			1	0.0852	0.0257
1 WPI	mean difference				0	-0.0155
	p-value				1	0.9782
5 WPI	mean difference					0
	p-value					1

Supplementary Table.S2.C. Epicentre Dorsal corticospinal tract Area, One-way ANOVA. Relates to Fig.5.C.

ANOVA table	sum-of-squares	DF	mean-squares	F (DFn, DFd)	P value
Time point	0.09218	4	0.02305	F (4, 22) = 1191	P<0.0001
Spinal Cord Replicates	0.0004256	22	0.00001934		
Total	0.09261	26			

		SHAM	24 HPI	72 HPI	1 WPI	5 WPI
SHAM	mean difference	0	-0.1859	-0.1859	-0.1859	-0.186
	p-value	1	<0.0001	<0.0001	<0.0001	<0.0001
24 HPI	mean difference		0	0.0000	0.0000	-0.0001
	p-value		1	>0.9999	>0.9999	>0.9999
72 HPI	mean difference			0	0.0000	0.0000
	p-value			1	>0.9999	>0.9999
1 WPI	mean difference				0	0.0000
	p-value				1	>0.9999
5 WPI	mean difference					0
	p-value					1

Supplementary Table.S2.D. Caudal Dorsal corticospinal tract Area, One-way ANOVA. Relates to Fig.5.D.

ANOVA table	sum-of-squares	DF	mean-squares	F (DFn, DFd)	P value
Time point	0.0221	4	0.005525	F (4, 22) = 5.070	P=0.0048
Spinal Cord Replicates	0.02398	22	0.00109		
Total	0.04608	26			

		SHAM	24 HPI	72 HPI	1 WPI	5 WPI
SHAM	mean difference	0	0.02089	0.05186	-0.01989	-0.02004
	p-value	1	0.8958	0.2088	0.9111	0.9088
24 HPI	mean difference		0	0.03097	-0.04077	-0.04093
	p-value		1	0.498	0.2396	0.2363
72 HPI	mean difference			0	-0.07174	-0.07190
	p-value			1	0.0085	0.0084
1 WPI	mean difference				0	-0.00016
	p-value				1	>0.9999
5 WPI	mean difference					0
	p-value					1

Supplementary Table.S2.E. Fasciculus gracilis area, Two-way RM-ANOVA. Relates to Fig.5.E.

ANOVA table	sum-of-squares	DF	mean-squares	F (DFn, DFd)	P value
Interaction	0.508279	84	0.00605094	F (84, 462) = 3.687217	P<0.0001
Rostro-caudal distance	1.640042	21	0.07809722	F (21, 462) = 47.58954	P<0.0001
Time point	0.9947957	4	0.2486989	F (4, 22) = 34.11307	P<0.0001
Spinal Cords	0.1603894	22	0.007290428	F (22, 462) = 4.442516	P<0.0001
Residual	0.7581691	462	0.001641059		

		SHAM	24 HPI	72 HPI	1 WPI	5 WPI
SHAM	mean difference	0	0.1031	0.2443	0.2516	0.3397
	p-value	1	0.1922	<0.0001	<0.0001	<0.0001
24 HPI	mean difference		0	-0.7270	-0.8294	-1.504
	p-value		1	<0.0001	<0.0001	<0.0001
72 HPI	mean difference			0	-0.1025	-0.7773
	p-value			1	0.9561	<0.0001
1 WPI	mean difference				0	-0.0881
	p-value				1	0.4092
5 WPI	mean difference					0
	p-value					1

Supplementary Table.S2.F. Rostral Fasciculus gracilis area, One-way ANOVA. Relates to Fig.5.F.

ANOVA table	sum-of-squares	DF	mean-squares	F (DFn, DFd)	P value
Time point	0.05045	4	0.01261	F (4, 22) = 12.96	P<0.0001
Spinal Cord Replicates	0.02141	22	0.0009732		
Total	0.07186	26			

		SHAM	24 HPI	72 HPI	1 WPI	5 WPI
SHAM	mean difference	0	-0.04148	-0.01319	-0.08461	-0.11910
	p-value	1	0.3564	0.974	0.0072	0.0002
24 HPI	mean difference		0	0.02829	-0.04314	-0.07766
	p-value		1	0.5303	0.1541	0.0024
72 HPI	mean difference			0	-0.07143	-0.10590
	p-value			1	0.0053	<0.0001
1 WPI	mean difference				0	-0.03452
	p-value				1	0.3382
5 WPI	mean difference					0
	p-value					1

Supplementary Table.S2.G. Epicentre Fasciculus gracilis area, One-way ANOVA. Relates to Fig.5.G.

ANOVA table	sum-of-squares	DF	mean-squares	F (DFn, DFd)	P value
Time point	0.1412	4	0.03529	F (4, 22) = 40.63	P<0.0001
Spinal Cord Replicates	0.01911	22	0.0008686		
Total	0.1603	26			

		SHAM	24 HPI	72 HPI	1 WPI	5 WPI
SHAM	mean difference	0	-0.16390	-0.20890	-0.25190	-0.21560
	p-value	1	<0.0001	<0.0001	<0.0001	<0.0001
24 HPI	mean difference		0	-0.04503	-0.08800	-0.05177
	p-value		1	0.0958	0.0003	0.0426
72 HPI	mean difference			0	-0.04297	-0.00674
	p-value			1	0.121	0.9944
1 WPI	mean difference				0	0.03623
	p-value				1	0.2435
5 WPI	mean difference					0
	p-value					1

Supplementary Table.S2.H. Caudal Fasciculus gracilis area, One-way ANOVA. Relates to Fig.5.H.

ANOVA table	sum-of-squares	DF	mean-squares	F (DFn, DFd)	P value
Time point	0.01133	4	0.002833	F (4, 22) = 1.733	P=0.1787
Spinal Cord Replicates	0.03596	22	0.001634		
Total	0.04729	26			

		SHAM	24 HPI	72 HPI	1 WPI	5 WPI
SHAM	mean difference	0	-0.01112	-0.00761	-0.05040	-0.04591
	p-value	1	0.9948	0.9988	0.4188	0.5093
24 HPI	mean difference		0	0.00351	-0.03928	-0.03479
	p-value		1	0.9999	0.4642	0.579
72 HPI	mean difference			0	-0.04279	-0.03830
	p-value			1	0.3808	0.4886
1 WPI	mean difference				0	0.00449
	p-value				1	0.9997
5 WPI	mean difference					0
	p-value					1

Supplementary Table.S2.I. Fasciculus cuneatus area, Two-way RM-ANOVA. Relates to Fig.5.I.

ANOVA table	sum-of-squares	DF	mean-squares	F (DFn, DFd)	P value
Interaction	5.168021	84	0.06152406	F (84, 462) = 4.624042	P<0.0001
Rostro-caudal distance	29.03874	21	1.382797	F (21, 462) = 103.9287	P<0.0001
Time point	2.314635	4	0.5786588	F (4, 22) = 6.522582	P=0.0013
Spinal Cords	1.951757	22	0.08871621	F (22, 462) = 6.667757	P<0.0001
Residual	6.147028	462	0.01330526		

		SHAM	24 HPI	72 HPI	1 WPI	5 WPI
SHAM	mean difference	0	-0.0050	0.1014	0.0553	0.1609
	p-value	1	>0.9999	0.1961	0.7334	0.0129
24 HPI	mean difference		0	-0.1064	-0.0603	-0.1659
	p-value		1	0.0571	0.4858	0.0014
72 HPI	mean difference			0	0.0461	-0.0594
	p-value			1	0.7188	0.5
1 WPI	mean difference				0	-0.1055
	p-value				1	0.0601
5 WPI	mean difference					0
	p-value					1

Supplementary Table.S2.J. Rostral Fasciculus cuneatus area, One-way ANOVA. Relates to Fig.5.J.

ANOVA table	sum-of-squares	DF	mean-squares	F (DFn, DFd)	P value
Time point	0.1201	4	0.03003	F (4, 22) = 5.474	P=0.0033
Spinal Cord Replicates	0.1207	22	0.005486		
Total	0.2408	26			

		SHAM	24 HPI	72 HPI	1 WPI	5 WPI
SHAM	mean difference	0	0.1159	0.1169	0.06622	-0.04915
	p-value	1	0.2123	0.2051	0.7148	0.8788
24 HPI	mean difference		0	0.0011	-0.04963	-0.165
	p-value		1	>0.9999	0.7729	0.0068
72 HPI	mean difference			0	-0.0507	-0.1661
	p-value			1	0.7594	0.0065
1 WPI	mean difference				0	-0.1154
	p-value				1	0.0866
5 WPI	mean difference					0
	p-value					1

Supplementary Table.S2.K. Epicentre Fasciculus cuneatus area, One-way ANOVA. Relates to Fig.5.K.

ANOVA table	sum-of-squares	DF	mean-squares	F (DFn, DFd)	P value
Time point	0.6802	4	0.1701	F (4, 22) = 13.78	P<0.0001
Spinal Cord Replicates	0.2715	22	0.01234		
Total	0.9517	26			

		SHAM	24 HPI	72 HPI	1 WPI	5 WPI
SHAM	mean difference	0	-0.33630	-0.50920	-0.50960	-0.47480
	p-value	1	0.0025	<0.0001	<0.0001	<0.0001
24 HPI	mean difference		0	-0.17280	-0.17320	-0.13850
	p-value		1	0.0872	0.0861	0.232
72 HPI	mean difference			0	-0.00043	0.03436
	p-value			1	>0.9999	0.9826
1 WPI	mean difference				0	0.03479
	p-value				1	0.9817
5 WPI	mean difference					0
	p-value					1

Supplementary Table.S2.L. Caudal Fasciculus cuneatus area, One-way ANOVA. Relates to Fig.5.L.

ANOVA table	sum-of-squares	DF	mean-squares	F (DFn, DFd)	P value
Time point	0.1958	4	0.04896	F (4, 22) = 9.218	P=0.0002
Spinal Cord Replicates	0.1168	22	0.005311		
Total	0.3127	26			

		SHAM	24 HPI	72 HPI	1 WPI	5 WPI
SHAM	mean difference	0	0.2164	0.2123	0.1746	0.03699
	p-value	1	0.0031	0.0037	0.02	0.9501
24 HPI	mean difference		0	-0.0041	-0.04174	-0.1794
	p-value		1	>0.9999	0.856	0.0026
72 HPI	mean difference			0	-0.03767	-0.1753
	p-value			1	0.8956	0.0033
1 WPI	mean difference				0	-0.1376
	p-value				1	0.026
5 WPI	mean difference					0
	p-value					1
